# M^6^A modification in lipid metabolism and related diseases

**DOI:** 10.3389/fgene.2026.1890451

**Published:** 2026-10-01

**Authors:** Zhiwen Hu, Didi Yuan, Xi Feng, Sai Hu

**Affiliations:** 1 Emergency Department, The Fourth Hospital of Changsha (Integrated Traditional Chinese and Western Medicine Hospital of Changsha, Changsha Hospital of Hunan Normal University), Changsha, Hunan, China; 2 Cancer Research Institute, Basic School of Medicine, Central South University, Changsha, Hunan, China

**Keywords:** atherosclerosis, lipid metabolism, M^6^A, non-alcoholic fatty liver disease, obesity

## Abstract

This review provides a systematic overview of the specific mechanisms by which N6-methyladenosine (m^6^A) modification regulates lipid synthesis, catabolism, and transport. In addition, we discuss the potential therapeutic significance of m^6^A modification in lipid metabolism-related diseases. Although considerable progress has been made, the mechanisms by which m^6^A modification regulates lipid metabolism remain incompletely understood. Notably, m^6^A regulators such as METTL3 exhibit context-dependent and sometimes opposing effects on lipid accumulation and oxidation. FTO gene variants are significantly associated with an increased risk of obesity; however, the development of FTO inhibitors as therapeutic targets for obesity and lipid metabolism disorders still faces considerable challenges. In non-alcoholic fatty liver disease (NAFLD), m^6^A modification not only contributes to disease development but also participates in its progression. METTL3 and METTL14 also play important roles in the development of atherosclerosis (AS). Collectively, the important role of m^6^A regulatory mechanisms in lipid metabolism and related diseases highlights the potential of targeting m^6^A regulators as a promising therapeutic strategy.

## 1 Introduction

Lipids are essential for maintaining cellular energy homeostasis and normal cellular function and play fundamental roles in human physiology and health. In addition to serving as major energy substrates and structural components of cellular membranes, lipids act as precursors for a wide range of bioactive molecules with important physiological functions (Petrenko et al., 2023). Lipid metabolism encompasses the synthesis, catabolism, and transport of fatty acids, cholesterol, and triglycerides (Chandel, 2021). Dysregulation of lipid metabolism is closely associated with various metabolic disorders, including obesity, nonalcoholic fatty liver disease, atherosclerosis, type 2 diabetes mellitus, hyperlipidemia, and cardiovascular disease (Pirillo et al., 2021). Overnutrition resulting from unhealthy dietary patterns and lifestyle factors is an important contributor to abnormalities in lipid metabolism. Given the increasing prevalence of these metabolic disorders, there is an urgent need to better understand the molecular mechanisms underlying the regulation of lipid metabolism (Roth et al., 2020).

Epigenetic regulation encompasses a range of mechanisms that modulate gene expression without altering the underlying DNA sequence, including DNA methylation, histone modifications, chromatin remodeling, and RNA-mediated regulation (Allis and Jenuwein, 2016). Among RNA modifications, m^6^A has emerged as an important post-transcriptional regulatory mechanism. M^6^A is the most prevalent internal modification of adenosine in eukaryotic RNA and plays a critical role in post-transcriptional gene regulation. M^6^A modification has been identified in a variety of RNA species, including messenger RNAs (mRNAs), transfer RNAs (tRNAs), ribosomal RNAs (rRNAs), circular RNAs (circRNAs), microRNAs (miRNAs), and long non-coding RNAs (lncRNAs). By modulating RNA splicing, stability, localization, translation, and secondary or higher-order structure, m^6^A modification contributes to diverse aspects of RNA metabolism and gene regulation (Zaccara et al., 2019). Increasing evidence indicates that dysregulated m^6^A modification is involved in the pathogenesis of various human diseases, including nonalcoholic fatty liver disease (Qin et al., 2021), azoospermia (Li G. et al., 2024), heart failure (Devaux and Nossent, 2020), and cancer (An and Duan, 2022).

This review summarizes recent advances in our understanding of the roles of m^6^A modification in lipid synthesis, transport, and catabolism. We focus on emerging evidence regarding the biological functions and underlying molecular mechanisms of m^6^A regulators in lipid metabolism and their involvement in lipid metabolism-related diseases. We also discuss the potential of m^6^A-related regulatory pathways as therapeutic targets for lipid metabolic disorders and associated diseases.

## 2 M^6^A modification

### 2.1 M^6^A modification-related enzymes

The m^6^A modification is the most prevalent internal modification found in eukaryotic mRNAs. On average, approximately 1–2 N6-methyladenosine residues are present per 1,000 nucleotides. M^6^A sites are preferentially enriched near stop codons, within 3′-untranslated regions (3′-UTRs), and in long intronic regions, and are primarily located within the conserved RRACH motif (R = A or G; H = A, C, or U) (Wang K. et al., 2021). The dynamic regulation of m^6^A modification is mediated by three major groups of regulatory proteins: methyltransferases (Writers), demethylases (Erasers), and m^6^A-binding proteins (Readers) (Meyer and Jaffrey, 2014). Table 1 summarizes the currently identified m^6^A regulators and their respective functions.

**TABLE 1:** M^6^A modification related enzymes and their functions.

Typology	m^6^A Enzyme	Functionality	Reference
Writers	METTL3	catalytic m^6^A modification	Su et al. (2024)
	METTL14	catalytic m^6^A modification and assisted METTL3 recognition	Quarto et al. (2025)
	METTL16	catalytic m^6^A modification	Sun et al. (2023)
	WTAP	promoting METTL3-METTL14 heterodimer formation	Lan et al. (2022)
	KIAA1429	specific recognition of RNA methyl-modified regions	Zhang C et al. (2022)
	RBM15	binding and recruitment of the m^6^A complex to specialized RNA sites	Park et al. (2024)
	ZC3H13	linking WTAP to the mRNA binding factors	Jia et al. (2011)
Erasers	FTO	remove m^6^A modifier	Chang et al. (2025)
	ALKBH5	remove m^6^A modifier	Huang et al. (2023)
Readers	YTHDC1	promotes RNA splicing and nucleation	Roundtree et al. (2017)
	YTHDC2	promotes mRNA translation	Mao et al. (2019)
	YTHDF1	promotes mRNA translation and stability	Rong et al. (2023a)
	YTHDF2	reduced mRNA stability	Wang and Lu (2021)
	YTHDF3	regulates mRNA translation and degradation	Chang et al. (2020)
	HNRNPA2B1	promotes primary microRNA processing	Alarcón et al. (2015)
	HNRNPC	mediates mRNA splicing	Huang et al. (2021)
	IGF2BP1/2/3	promotes mRNA stability	Ramesh-Kumar and Guil (2022)

Methyltransferase-like 3 (METTL3) is the catalytic subunit of the major m^6^A methyltransferase complex, whereas methyltransferase-like 14 (METTL14) is a homologous protein that forms a heterodimer with METTL3. Wilms tumor 1-associated protein (WTAP) recruits and stabilizes the METTL3–METTL14 complex, thereby facilitating its m^6^A methyltransferase activity and regulating global m^6^A levels (Patil et al., 2016). Methyltransferase-like 16 (METTL16) participates not only in m^6^A modification of conventional RNA substrates but also in the modification of specific RNA species, including small nuclear RNAs (snRNAs) and small nucleolar RNAs (snoRNAs) (Zhang H. et al., 2023). RNA-binding motif protein 15 (RBM15), zinc finger CCCH-type containing 13 (ZC3H13), and KIAA1429 (also known as VIRMA) can cooperate with other components of the m^6^A methyltransferase machinery to regulate RNA methylation and gene expression by recognizing or recruiting the complex to specific RNA sites (Zhang et al., 2022a; Jiang et al., 2022).

Fat mass and obesity-associated protein (FTO) was the first m^6^A demethylase to be identified (Jia et al., 2011). FTO removes m^6^A from a broad range of RNA substrates in an α-ketoglutarate (α-KG) - and Fe (II)-dependent manner. FTO initially oxidizes m^6^A to the intermediate N6-hydroxymethyladenosine (hm6A), which is further oxidized to N6-formyladenosine (f6A). These intermediates subsequently undergo spontaneous decomposition, ultimately regenerating adenosine and thereby resulting in m^6^A demethylation (Huang et al., 2023). AlkB homolog 5 (ALKBH5) is another well-characterized m^6^A demethylase that efficiently removes m^6^A from RNA (Liao et al., 2024), with a catalytic mechanism similar to that of FTO.

M^6^A modifications exert their biological functions through recognition by specific RNA-binding domains and proteins. The YTH domain, named after its conserved tyrosine and histidine residues, is a well-characterized structural module capable of directly recognizing m^6^A-modified RNA. YTH domain-containing (YTHDC) and YTH domain family (YTHDF) proteins constitute the major YTH domain-containing m^6^A readers, including YTHDC1, YTHDC2, YTHDF1, YTHDF2, and YTHDF3 (Chen L. et al., 2023; Cipriano et al., 2020). In addition, members of the heterogeneous nuclear ribonucleoprotein (HNRNP) family, including HNRNPA2B1 and HNRNPC, have been reported to recognize m^6^A-modified RNAs and participate in post-transcriptional regulation (Wang J. et al., 2021). Insulin-like growth factor 2 mRNA-binding proteins (IGF2BPs) constitute another class of RNA-binding proteins that recognize m^6^A-modified transcripts and regulate their stability and translation (Ramesh-Kumar and Guil, 2022). With increasing understanding of RNA modifications, these proteins have been recognized as important components of the m^6^A reader system.

### 2.2 M^6^A modification mechanism of action

The fundamental process of m^6^A modification involves the post-transcriptional regulation of RNA through methylation by methyltransferases (Writers), demethylation by demethylases (Erasers), and recognition by m^6^A-binding proteins (Readers). M^6^A modification plays a pivotal role in various aspects of RNA metabolism, including translation, degradation, splicing, nuclear export, and RNA folding (Zaccara et al., 2019). By influencing these processes, m^6^A modification contributes to determining RNA fate and regulates a broad range of mRNA biological functions (Figure 1) (Wang et al., 2014).

**FIGURE 1:**
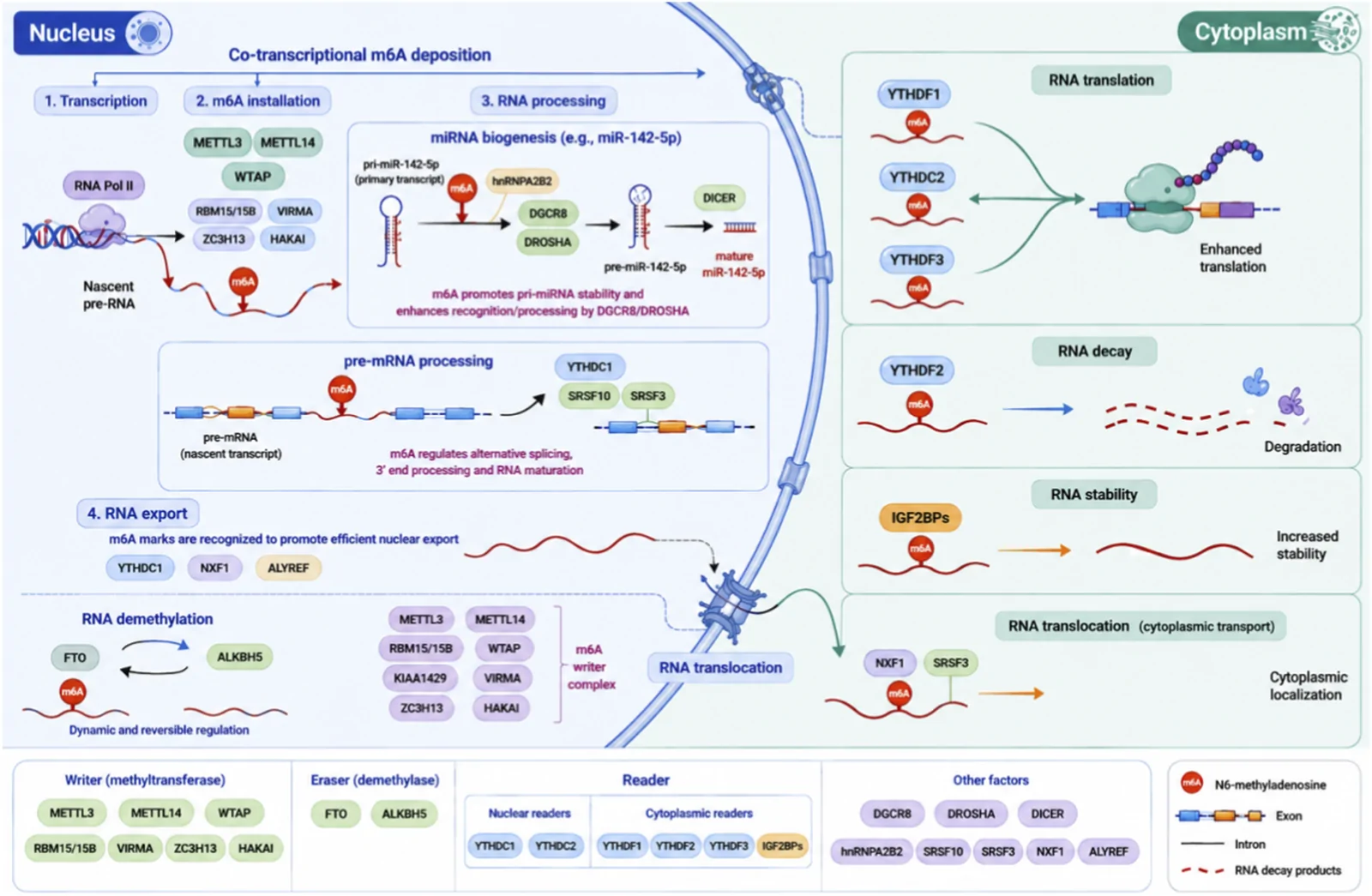
Overview of m^6^A-mediated regulation of RNA processing and fate in the nucleus and cytoplasm. M^6^A is dynamically regulated by methyltransferases, demethylases, and RNA-binding readers. In the nucleus, m^6^A deposition on nascent RNA contributes to miRNA biogenesis, mRNA processing, and pre-mRNA splicing. In particular, m^6^A modification of pri-miRNAs can facilitate their recognition and processing by the DGCR8–DROSHA machinery, thereby promoting miRNA maturation. Nuclear m^6^A readers, including YTHDC1, together with associated splicing factors, participate in RNA processing and splicing. M^6^A-marked RNAs can subsequently undergo nuclear export and enter the cytoplasm, where cytoplasmic readers regulate RNA translation, stability, decay, and localization. Thus, m^6^A provides a dynamic post-transcriptional regulatory layer that connects RNA processing in the nucleus with RNA fate in the cytoplasm.

M^6^A modification is a tightly regulated process. The m^6^A level is partly determined by the intrinsic sequence preference of the METTL3 / METTL14 methyltransferase complex and is also influenced by additional factors that modulate the activity or recruitment of the methyltransferase complex, including RNA-binding proteins, transcription factors, RNA polymerases, and other regulatory factors. Furthermore, different m^6^A readers can compete for m^6^A-modified transcripts or modification sites, thereby influencing the stability, translation, and other biological fates of target RNAs.

YTHDF2 selectively recognizes m^6^A-modified RNAs through multiple mechanisms and promotes their degradation (Wang and Lu, 2021). YTHDF2 recruits the CCR4-NOT deadenylase complex to facilitate deadenylation and subsequent degradation of m^6^A-modified mRNAs. In addition, YTHDF2 can recruit the RNase P/MRP complex, thereby contributing to the degradation of m^6^A-modified mRNA molecules (Du et al., 2016). Conversely, RNA-binding proteins IGF2BP1, IGF2BP2, and IGF2BP3 can counteract this process by recognizing and stabilizing m^6^A-modified mRNAs, particularly within the 3′-UTRs of target transcripts (Ramesh-Kumar and Guil, 2022). Notably, limited overlap has been observed between the transcriptome-wide binding sites of IGF2BP1–3 and YTHDF2, suggesting that m^6^A sites may be differentially recognized by IGF2BP1–3 or YTHDF2, thereby leading to distinct effects on RNA stability and fate.

The regulation of mRNA translation by m^6^A modification depends on the selective recognition of m^6^A sites by m^6^A readers. YTHDF1 and YTHDF3 promote translation by facilitating the recruitment of translation initiation factor eIF3 to mRNAs (Liu et al., 2020). In addition, YTHDC2 has been shown to enhance translation elongation by facilitating the resolution of RNA secondary structures (Mao et al., 2019). Studies from Junho Choe’s group demonstrated that the METTL3–eIF3h axis mediates mRNA recycling and translation by facilitating the interaction of METTL3 with a specific region near the translation termination codon through the eIF3h subunit. Moreover, m^6^A modification at this site can directly interact with eIF3 to enhance mRNA translation. M^6^A sites located near the stop codon and within the 3′-UTR can also be recognized by YTHDF1 and YTHDF3, thereby promoting translation initiation (Choe et al., 2018).

Moreover, m^6^A modification regulates mRNA splicing, processing, and nuclear export. The m^6^A reader YTHDC1 directly modulates alternative splicing by recruiting SRSF3 while inhibiting the binding of SRSF10 to target RNAs (Xiao et al., 2016). HNRNPA2B1 regulates selective exon splicing in various transcripts in a METTL3-dependent manner. In addition, HNRNPA2B1 interacts with DGCR8, a component of the pri-miRNA microprocessor complex, thereby facilitating pri-miRNA processing (Alarcón et al., 2015). In HeLa cells, knockdown of YTHDC1 resulted in increased fluorescence *in situ* hybridization signals in the nucleus and reduced signals in the cytoplasm, suggesting that YTHDC1 positively regulates nuclear mRNA export, potentially by facilitating the interaction of mRNA with SRSF3 and NXF1 (Roundtree et al., 2017).

## 3 M^6^A modification and lipid metabolis

### 3.1 M^6^A modification and lipid synthesis and catabolism

Lipid metabolism encompasses the processes of lipid digestion, absorption, synthesis, and catabolism. Among these processes, the balance between lipid synthesis and catabolism is essential for maintaining cellular lipid homeostasis and overall metabolic health. Acetyl-CoA carboxylase (ACC), fatty acid synthase (FASN), and stearoyl-CoA desaturase 1 (SCD1) are key enzymes involved in fatty acid synthesis. Peroxisome proliferator-activated receptor α (PPARα) is a key regulator of fatty acid oxidation and plays an important role in lipid catabolism. Sterol regulatory element-binding proteins (SREBPs), including SREBP-1a, SREBP-1c, and SREBP-2, are important transcription factors involved in lipid metabolism, with SREBP-1c playing a particularly prominent role in fatty acid synthesis. M^6^A modification contributes to the regulation of lipid metabolism by modulating the expression and function of lipid metabolism-related molecules. Figure 2 illustrates the mechanisms through which m^6^A modification regulates lipid metabolism.

**FIGURE 2:**
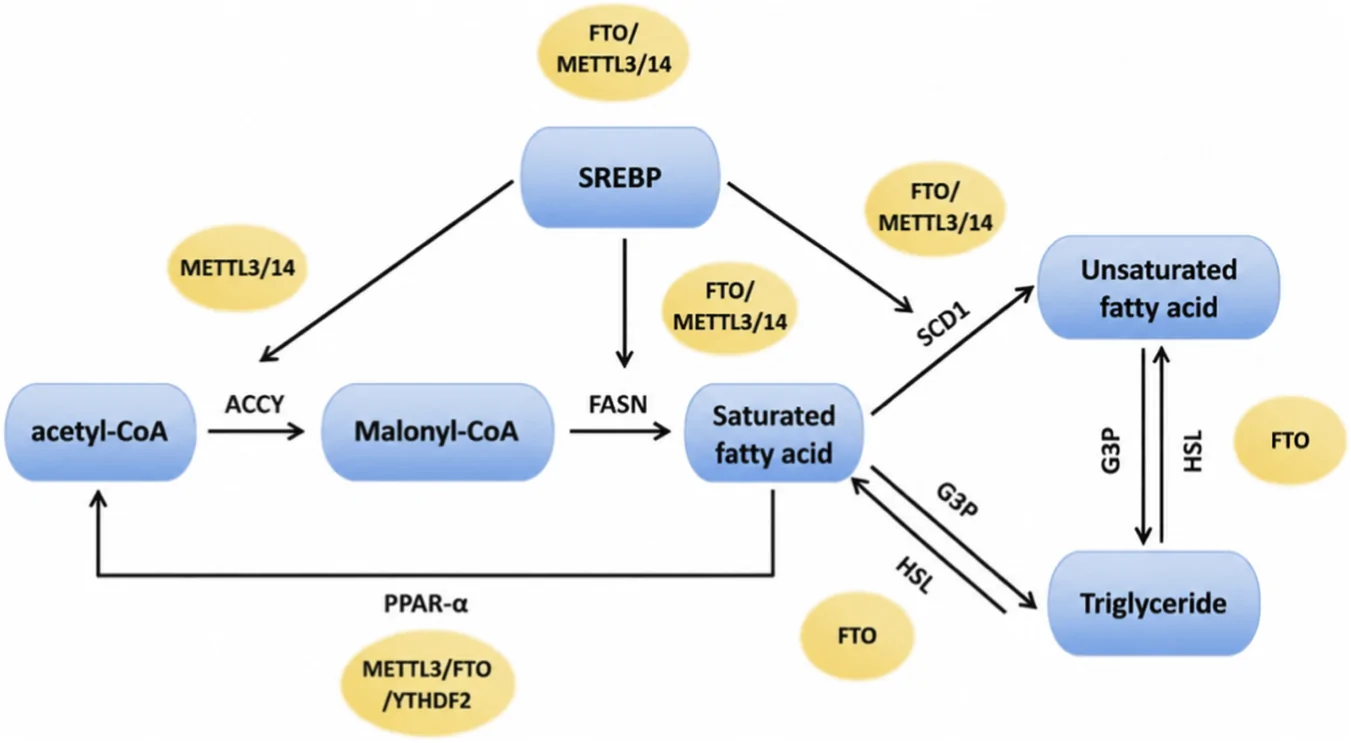
Triglyceride Synthesis and Degradation and Methylation Regulation. Acetyl-CoA: Acetyl-Coenzyme A; Malonyl-CoA: Malonyl-Coenzyme A; ACCY: Acetyl-CoA Carboxylase; FASN: Fatty acid Synthase; Fatty acid: Saturated fatty acids; SCD1: Stearoyl-CoA Desaturase 1; G3P: Glycerol-3-phosphate; HSL: Hormone-Sensitive Lipase; PPAR-α: Peroxisome Proliferator-Activated Receptor α; SREBP: Sterol Regulatory Element-Binding Protein; yellow represents the m^6^A enzyme; blue represents molecules related to lipid metabolism. The regulatory effects of m^6^A modification are dependent on specific m^6^A regulators, target transcripts, and cellular contexts.

M^6^A modification may exert protective effects against lipid accumulation in certain contexts. Studies have shown that depletion of the methyltransferase complex components METTL14 or METTL3 increases the levels of FASN, SCD1, and ACC in the liver of rodents, leading to enhanced triglyceride (TG) production and increased liver weight. Consistently, *in vitro* depletion of METTL3 or METTL14 increased ACC and SCD1 protein levels, enhanced triglyceride and cholesterol production, and promoted lipid droplet accumulation (Yang Y. et al., 2022). FTO was the first m^6^A demethylase to be identified. FTO can bind to the 3′-UTRs of various adipogenic genes and stabilize their mRNAs by reducing m^6^A modification levels (Chang et al., 2025). Reported targets of FTO include SCD1, PPARα, and SREBP. FTO has been shown to increase SREBP-1c expression by reducing its m^6^A modification level, thereby promoting the expression of lipid metabolic genes, including FASN, SCD1, ACC, and ACCα, and increasing lipid accumulation (Yang Z. et al., 2022). In addition, FTO has been reported to regulate triglyceride metabolism by modulating hormone-sensitive lipase (HSL)-mediated lipolysis (Jiao et al., 2016).

M^6^A modification also contributes to the regulation of lipid catabolism and fatty acid oxidation. METTL3 promotes m6A modification of PPARα mRNA, which is recognized by the m6A reader YTHDF2, thereby promoting PPARα mRNA degradation and reducing PPARα expression, ultimately affecting hepatic lipid metabolism (Zhong et al., 2018). Angiopoietin-like protein 4 (ANGPTL4) is an important regulator of triglyceride metabolism and can inhibit lipoprotein lipase (LPL), thereby modulating extracellular triglyceride hydrolysis. FTO has been reported to reduce ANGPTL4 mRNA translation, thereby promoting extracellular triglyceride hydrolysis (Yang Z. et al., 2022).

M^6^A modification may also regulate lipid metabolism by modulating cellular autophagy. FTO promotes autophagy and adipogenesis by reducing the m^6^A modification levels of the autophagy-related genes ATG5 and ATG7, thereby facilitating the YTHDF2-mediated degradation of ATG5 and ATG7 mRNAs in the cytoplasm (Wang X. et al., 2020). A recent study further demonstrated that METTL3 reduces lipid accumulation in an atherosclerosis (AS) mouse model by promoting the stability of AKT1S1 and activating autophagy (Yu et al., 2024). In addition, FTO has been reported to reduce adipocyte apoptosis by inhibiting the mitochondrial unfolded protein response and activating the STAT3/JAK2 signaling pathway (Shen et al., 2021).

In recent years, increasing attention has been given to the roles of m^6^A readers in lipid metabolism. YTHDF2 has been reported to increase blood cholesterol levels by promoting the translation of 6-phosphogluconate dehydrogenase (6PGD) mRNA (Sheng et al., 2020). In addition, YTHDF2 plays an important role in regulating triglyceride homeostasis and adipogenesis in NAFLD by modulating the expression of SREBP1c, FASN, SCD1, and ACC (Wang and Lu, 2021). These findings highlight the potential of m^6^A readers as therapeutic targets for the regulation of lipid metabolism and reduction of blood cholesterol levels.

### 3.2 Regulation of lipid transport

Lipid transport is a dynamic process governed by both intrinsic membrane properties and active regulatory mechanisms. Although lipids exhibit considerable lateral mobility within membrane bilayers, their transbilayer movement (flip-flop) is relatively restricted and is regulated by specialized lipid transport mechanisms, membrane contact sites, vesicular trafficking, and membrane remodeling processes (Voeltz et al., 2024). Multiple classes of proteins participate in lipid transport, including membrane lipid transporters such as ATP-binding cassette (ABC) transporters (e.g., ABCA1 and ABCG1), plasma lipid transport proteins such as apolipoproteins (APOA, APOB, and APOE), and intracellular cholesterol transport proteins such as Niemann–Pick disease type C proteins (NPC1 and NPC2). Emerging evidence suggests that m^6^A modification may regulate lipid transport primarily by modulating the expression, stability, and translation of transcripts encoding lipid transport-related proteins, thereby indirectly influencing their abundance and activity.

METTL3-mediated m^6^A modification appears to exert context-dependent effects on lipid transport. Mettl3-mediated hypermethylation of NPC1L1 mRNA promotes lipid accumulation through regulation of the MAPK pathway (Zhang G. et al., 2023). In METTL3-knockout mice, ABCG8 was significantly hypomethylated and downregulated, resulting in reduced cholesterol excretion. FTO has also been implicated in the regulation of lipid transport. FTO-mediated demethylation inhibits lipid uptake by macrophages by reducing PPARγ protein expression and promotes cholesterol efflux through AMPK phosphorylation, thereby suppressing foam cell formation and the development of atherosclerosis (Yang Z. et al., 2022). Recent evidence further indicates that FTO overexpression increases APOE expression in an m^6^A–IGF2BP2-dependent manner, thereby contributing to lipid metabolic dysregulation in tumors (Chen et al., 2025). Similarly, ALKBH5 overexpression increases FABP5 expression in an m^6^A–IGF2BP2-dependent manner, thereby influencing intracellular fatty acid utilization and storage (Chen et al., 2023a). In addition, YTHDF2 regulates cholesterol homeostasis by recognizing m^6^A modification on low-density lipoprotein receptor (LDLR) mRNA and promoting its degradation (Chen L. et al., 2023). Collectively, these findings suggest that m^6^A-mediated post-transcriptional regulation may influence lipid transport by modulating the expression, stability, and translation of transcripts encoding lipid transport-related proteins. However, the extent to which m^6^A modification directly or indirectly contributes to the regulation of lipid transport remains largely unclear and warrants further investigation.

Although accumulating evidence indicates that m^6^A modification is involved in lipid metabolism, the functions of individual m^6^A regulators remain highly context-dependent and may even be contradictory across different experimental settings. For example, METTL3 and METTL14 have been reported to either promote or suppress lipid accumulation (Yang Y. et al., 2022; Wu et al., 2020). Depletion of these methyltransferases may increase the expression of lipogenic genes, such as FASN and SCD1, whereas other studies have shown that METTL3-mediated m^6^A modification promotes fatty acid oxidation through metabolic regulators such as PPARα (An and Duan, 2022). Similarly, FTO has been implicated in both lipid accumulation and lipid homeostasis through its effects on lipogenic pathways and fatty acid oxidation. These apparent discrepancies may arise from differences in tissue specificity, metabolic state, experimental models, target RNA selection, and the involvement of distinct m^6^A readers. Therefore, m^6^A regulators should not be broadly classified as either pro-lipogenic or anti-lipogenic factors. Rather, their effects on lipid metabolism are likely determined by complex interactions between cellular context, target transcripts, and epitranscriptomic regulatory mechanisms.

## 4 M^6^A modification and lipid metabolism-related diseases

Lipid metabolism disorders are closely related to a variety of diseases, including obesity, NAFLD, atherosclerosis, type 2 diabetes, and hyperlipidemia. Although these diseases vary in their tissue origin and clinical manifestations, they share several common characteristics. As shown in Figure 3, m^6^A regulatory factors may affect these interrelated metabolic processes by regulating the expression, stability, and translation of key lipid metabolism-related transcripts, thereby promoting the occurrence and development of the diseases in a tissue- and environment-dependent manner. The three representative diseases, obesity, NAFLD, and atherosclerosis, cover multiple aspects of lipid metabolism imbalance that are interrelated yet distinct, ranging from lipid storage and remodeling in adipose tissue, to liver lipid accumulation, and cholesterol deposition in blood vessels. Therefore, the following section will explore the emerging roles of m^6^A regulatory factors in these diseases.

**FIGURE 3:**
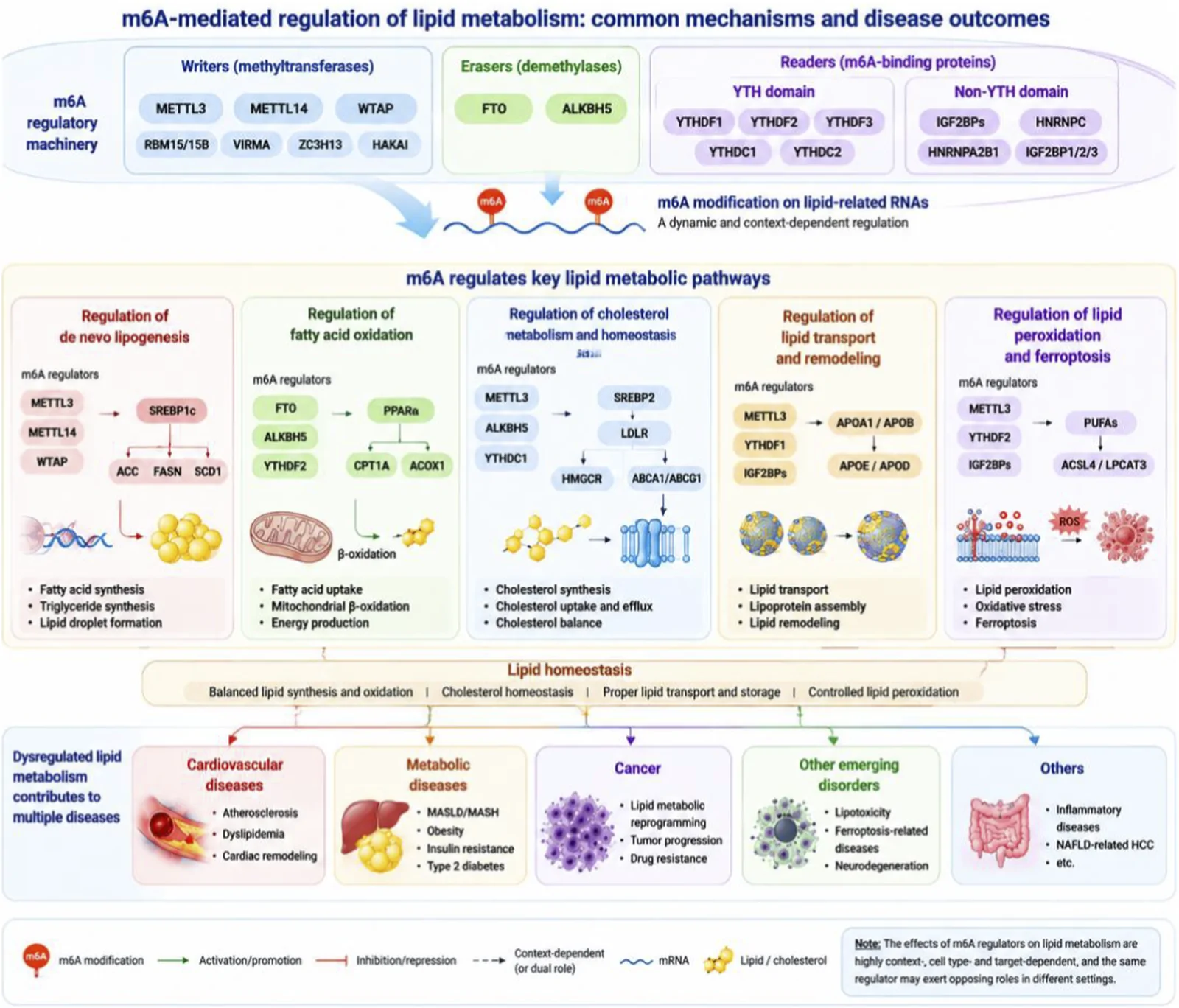
Integrated framework linking m^6^A regulation to lipid metabolism and lipid-associated diseases. Dysregulation of m^6^A modification can influence lipid homeostasis through the coordinated actions of m^6^A writers, erasers, and readers on lipid metabolism-related transcripts. These regulatory mechanisms converge on several interconnected metabolic processes, including *de novo* lipogenesis, fatty acid oxidation, cholesterol homeostasis, lipid transport and storage, and lipid peroxidation. Alterations in these pathways may lead to tissue- and context-dependent disturbances in lipid homeostasis and contribute to the development or progression of obesity, NAFLD, AS. The figure highlights the shared metabolic mechanisms linking m^6^A regulation to these representative lipid metabolism-related diseases, while also illustrating the disease-specific consequences of lipid metabolic dysregulation.

### 4.1 Obesity

Obesity is a metabolic disorder characterized by lipid overload, expansion of adipose tissue, and ectopic deposition of lipids. Globally, there is increasing evidence suggesting that obesity is associated with the remodeling of the m^6^A epigenetic landscape in adipose tissue. A recent comprehensive transcriptome m^6^A profiling study of human subcutaneous and visceral adipose tissues identified thousands of m^6^A-modified transcripts and revealed significant differences in methylation patterns between obese individuals and those of normal weight. Notably, these m^6^A remodeling events are closely related to clinical variables related to glucose and insulin metabolism, suggesting that changes in the m^6^A landscape in adipose tissue may be associated with metabolic remodeling in obesity (Ronningen et al., 2025). Table 2 summarizes the currently known m^6^A regulatory mechanisms related to obesity.

**TABLE 2:** Effects of m^6^A regulators on obesity through regulation of target transcript methylation and downstream molecular functions.

Regulator	Change	Target	Effect	Ref.
ALKBH5	↑	LCAT	Affects LCAT mRNA stability and reduces preadipocyte differentiation	Chao et al. (2024)
ALKBH5/METTL3/METTL14/ FTO/YTHDF2	-	CES2	Double knockdown of METTL3/METTL14 increased CES2 mRNA levels and decreased lipid accumulation, whereas knockdown of FTO/ALKBH5 did the opposite; YTHDC2 degraded CES2 mRNA and promoted lipid accumulation	Takemoto et al. (2021)
FTO	↓	Hif1α	YTHDC2 recognition facilitates mRNA translation	Wu et al. (2021)
FTO	↑	CCND1/CDK2	Reduced mRNA stability after YTHDF2 recognition	Liao et al. (2022)
FTO	↑	CPT1 CPT2	Decreased CPT1/CPT2 mRNA expression and increased hepatic triglyceride accumulation	Zhang et al. (2016)
FTO	↓	FASN	YTHDF2 promotes FASN mRNA degradation and inhibits *de novo* adipogenesis	Sun et al. (2021)
FTO	↑	Perilipin5	Increases Perilipin5 expression and promotes triglyceride metabolism	Wei et al. (2021)
FTO	↓	ATG5/ATG7	YTHDF2 recognition promotes ATG5/ATG7 mRNA degradation, attenuating autophagy and adipogenesis	Wang X et al. (2020)
FTO	↑	NADP	NADP directly binds to FTO and enhances its m6A demethylase activity, thereby promoting RNA m6A demethylation and adipogenesis	Wang L et al. (2020)
FTO	↓	CCNA2/CDK2	YTHDF2 recognizes and degrades CCNA2/CDK2 mRNA to prolong the cell cycle and inhibit adipogenesis	Wu et al. (2018a)
FTO	↑	ADRB1	Upregulation of ADRB1 gene expression inhibits preadipocyte differentiation	Luo et al. (2023)
FTO	↑	CTNNB1	Increased CTNNB1 mRNA stability promotes chicken preadipocytosis	Li K et al. (2022)
FTO	↓	JAK2	YTHDF2 accelerates JAK2 mRNA decay, leading to inactivation of JAK2- stat3 - c/EBP β signaling, thereby inhibiting adipogenesis	Wu R et al. (2019)
FTO	↓	CCNA2/CDK2	EGCG reduces FTO to promote YTHDF2 expression, targets CCNA2/CDK2, and inhibits adipogenesis	Wu et al. (2018b)
FTO	↑	HSP60	Reduces HSP60 expression and adipocyte apoptosis	Shen et al. (2021)
IGF2BP3	↓	MYLK	Shortens MYLK half-life and promotes lipidogenic differentiation	Huang et al. (2024)
IGF2BP2	-	Bim	Promotes Bim stability and induces mitochondrial apoptosis in adipocytes	Wang et al. (2024a)
METTL3	↑	JAK1	METTL3 targets the JAK1/STAT5/C/EBPβ pathway in an m^6^A -YTHDF2-dependent manner and inhibits BMSC lipogenic differentiation	Y et al. (2019)
METTL3	↑	CCND1	Deletion of ZFP217 increases METTL3 expression, which downregulates CCND1 expression, and lipogenesis is inhibited	Liu et al. (2019)
METTL3	↓	DDIT4	Increased DDIT4 mRNA levels improve obesity	Qin et al. (2021)
METTL3	↑	Lipid metabolism gene	IGF2BP2 recognition promotes mRNA stability and beige fat activation	Li et al. (2023a)
METTL14	↑	PTGES2/CBR1	Reduced PTGES2/CBR1 mRNA decay in brown fat	Xiao L et al. (2025)
METTL14	↑	TRAILT/NFR1	Reduces TRAILT/NFR1 mRNA decay in white adipose tissue	Xiao L et al. (2025)
METTL14	↑	Adrb2/3,Atgl,Cgi-58	Reduces translation and protein levels	Kang et al. (2023)
YTHDF1	-	PNPLA2	Promotes PNPLA2 mRNA translation and limits fat accumulation	Wang et al. (2018)
YTHDF1	-	MTCH2	m^6^A–YTHDF1-dependent regulation of MTCH2 promotes intramuscular preadipocyte adipogenesis	Jiang et al. (2019)
YTHDF2	-	FAM134B	Promotes FAM134B mRNA degradation and inhibits adipogenic differentiation	Cai et al. (2019)
YTHDF2	↓	circ-SLC9A6	Reduces circ-SLC9A6 degradation and impairs lipid metabolism	Wang et al. (2024b)

“—” indicates that no specific change in the expression level of the m^6^A regulator or no specific target was reported in the cited study; The arrows (↑ and ↓) indicate increases or decreases in the expression/activity of m^6^A regulators.

FTO was the first m^6^A regulator identified in association with lipid metabolism (). Studies have shown that FTO expression is positively correlated with body mass index (BMI) and body fat content in individuals with obesity. In addition, FTO gene polymorphisms have been closely associated with susceptibility to obesity. A genome-wide association study (GWAS) demonstrated that genetic variants in the FTO locus are associated with an increased risk of obesity, with the rs9939609 and rs17817449 variants showing significant associations with overweight and obesity (Ning et al., 2024). Furthermore, Zhou et al. reported that the rs34269950 polymorphism within an FTO recognition motif was significantly associated with waist circumference (Zhou et al., 2021). These findings suggest that FTO may represent a potential therapeutic target for obesity. In a recent study, an FTO degradation-targeting chimera (FTO-DT) was developed to selectively degrade FTO protein, thereby reducing lipid deposition in hepatocytes and adipocytes in mice fed a high-fat diet (Xiao Y. et al., 2025). However, the clinical translation of FTO-targeted therapies remains challenging. However, the clinical translation of FTO-targeted therapies remains challenging. Entacapone, a repurposed FTO inhibitor, has shown potential for improving metabolic phenotypes in animal models by inhibiting FTO-mediated m^6^A demethylation; however, its clinical application as an FTO-targeting agent remains limited due to insufficient pharmacological optimization, low potency, short plasma half-life, and the lack of clinical validation for metabolic diseases (Peng et al., 2019; Carletti et al., 2022). Genetic variation, interindividual differences, potential off-target effects, and the need for further optimization remain important challenges for therapeutic strategies targeting FTO in obesity and lipid metabolic disorders.

The mechanisms by which m^6^A modification regulates adipogenesis are complex and context-dependent. In an *in vitro* 3T3-L1 preadipocyte model, FTO was shown to regulate the exon splicing of the adipogenesis-related transcription factor RUNX1T1 by altering m^6^A abundance near the splicing site, thereby promoting lipid droplet formation in adipocytes (Zhao et al., 2014). In addition, the m^6^A methyltransferase complex comprising METTL3, METTL14, and WTAP has been reported to promote adipogenesis by regulating mitotic clonal expansion (MCE). Conversely, another *in vitro* study demonstrated that m^6^A modification could regulate lipid accumulation in porcine adipocytes independently of the potential effects of FTO and METTL3, with m^6^A levels showing a negative association with adipogenesis (Cai et al., 2019). Together, these findings indicate that the effects of m^6^A modification on adipogenesis may vary according to the cellular context, regulatory factors, and experimental model.

Recent studies have suggested that induction or activation of brown adipose tissue (BAT) may represent a potential therapeutic strategy for obesity. FTO-dependent m^6^A demethylation has been implicated in the regulation of white adipose tissue (WAT) browning. Following recognition by the m^6^A reader YTHDC2, m^6^A-modified HIF1α transcripts undergo enhanced translation. Activated HIF1α subsequently stimulates the expression of PPARGC1A, PRDM16, and PPARG, which collectively increase UCP1 expression and promote the browning of white adipocytes (Li et al., 2023a). Yan et al. reported that targeted downregulation of YTHDF1 in adipocytes of mice fed a high-fat diet (HFD) reduced BMP8B translation, thereby exacerbating obesity, insulin resistance (IR), and glucose intolerance and impairing WAT browning (Yan et al., 2023). In addition, Xie et al. demonstrated that the chemical ligand methylpiperidine-3-carboxylate can activate the METTL3 complex and promote WAT browning (Xie et al., 2023).

Given the important roles of genetic and epigenetic factors in determining birth weight and susceptibility to childhood obesity, parental obesity and dietary status may influence obesity risk in offspring through epigenetic mechanisms. Studies have shown that offspring of pregnant mice fed an HFD exhibited elevated m^6^A modification levels in visceral adipose tissue at 3 weeks of age, accompanied by downregulation of FTO and upregulation of METTL3 expression. However, at 8 weeks of age, both FTO and METTL3 transcript levels were significantly increased, whereas the overall m^6^A level remained unchanged (Izquierdo et al., 2021). Another study reported that offspring of parents consuming a low-fat diet exhibited alterations in body weight, metabolic parameters, and insulin resistance, accompanied by increased expression of METTL3, FTO, and hypothalamic CREB-binding protein (CREBBP) (Kaspi et al., 2018). These findings suggest that parental dietary status may influence metabolic phenotypes in offspring through epigenetic regulation and highlight the potential importance of parental dietary management in reducing the risk of obesity in subsequent generations.

### 4.2 NAFLD

Hepatic lipid metabolic dysfunction is a major feature of NAFLD. Emerging evidence suggests that dysregulated RNA methylation is associated with hepatic metabolic remodeling in NAFLD. RNA methylation immunoprecipitation sequencing and transcriptome sequencing of the livers of mice with high-fructose diet-induced NAFLD revealed widespread alterations in RNA methylation, with differentially methylated transcripts predominantly enriched in lipid metabolism-related pathways, suggesting a potential association between altered RNA methylation and hepatic lipid metabolic dysfunction (Luo et al., 2021). Table 3 summarizes the currently identified m^6^A regulatory mechanisms associated with NAFLD. In 2023, a multisociety consensus introduced the new nomenclature of metabolic dysfunction-associated steatotic liver disease (MASLD) and metabolic dysfunction-associated steatohepatitis (MASH), replacing the former terminology of NAFLD and non-alcoholic steatohepatitis (NASH) within the updated classification framework (Rinella et al., 2023).

**TABLE 3:** Effects of m^6^A regulators on NAFLD through regulation of target transcript methylation and downstream molecular functions.

Regulator	Change	Target	Effect	Ref.
FTO	↓	SLC7A11	Arbutin (ARB) alleviates NAFLD by modulating the FTO/SLC7A11 pathway, increasing SLC7A11 expression and suppressing ferroptosis	Jiang et al. (2023)
FTO	↑	SF-1/Stearoyl-CoA	Promotion of hepatic lipid accumulation (mechanism of glucocorticoid-induced lipid accumulation)	Hu et al. (2020)
FTO	↑	PGC1 α	Betaine promotes FTO expression, thereby upregulating PGC1α expression and inhibiting hepatic lipid accumulation	Liu et al. (2024)
FTO	↑	SREBF1/ChREBP	Increased stability of SREBF1 and ChREBP mRNA to promote hepatic steatosis	Tang et al. (2023)
METTL3	↓	DDIT4	Downregulation of DDIT4 mRNA levels improves NAFLD	Qin et al. (2021)
METTL3	↓	PPAR α	Increased longevity and expression of PPAR alpha mRNA reduces lipid accumulation in adipocytes *in vitro* (m^6^A oscillations are influenced by the biological clock)	Zhong et al. (2018)
METTL3	↑	CYP2B6	Promotes CYP2B6 translation levels and increases hepatocyte insulin resistance	Li et al. (2023b)
METTL3	↑	Rubicon	Interaction with YTHDF1 promotes Rubicon mRNA stability and blocks lipid droplet clearance	Peng et al. (2022)
METTL3	↓	DDIT4	Increased macrophage DDIT4 expression and significant downregulation of mTOR and NF-κB pathway activity improve NAFLD	Qin et al. (2021)
METTL3	↑	SCD1	YTHDF1 recognition increases SCD1 mRNA stability and promotes NAFLD progression	Meng et al. (2024)
METTL3	↑	ACSS3	RPN11 - METTL3 - ACSS3 - Histone propionylation pathway activation, hepatic lipid accumulation	Zhou B et al. (2024)
METTL14	↑	DGCR8	Regulation of the DGCR8/miR-34a-5p/SIDT2 axis impairs mitochondrial homeostasis in NAFLD	Wang et al. (2024c)
METTL14	↑	NLRP3	Enhanced stability of NLRP3 mRNA to promote arsenic-induced liver IR	Qiu et al. (2023)
METTL16	↑	CIDEA	Increased expression levels of the lipid gene CIDEA in HepG2 cells	Tang et al. (2022)
RBM15	↑	RNF5	Upregulation of RNF5 expression, RNF5 inhibits ROCK1 protein levels through ubiquitination and accelerates NAFLD	Li et al. (2024b)

The arrows (↑ and ↓) indicate increases or decreases in the expression/activity of m^6^A regulators.

M^6^A dysregulation can modulate hepatic lipid accumulation in a context-dependent manner. High-throughput sequencing and RNA sequencing analyses showed that METTL16 was significantly upregulated in an *in vitro* NAFLD model, and further studies indicated that METTL16 promoted lipid accumulation by enhancing the expression of the adipogenic gene CIDEA (Tang et al., 2022). In the liver of NAFLD mice, FTO-mediated demethylation increased the stability of the mRNAs encoding the lipogenic transcription factors sterol regulatory SREBF1 and ChREBP. Knockdown of either SREBF1 or ChREBP attenuated the lipogenic effects of FTO, whereas treatment with the FTO inhibitor entacapone reduced the expression of SREBF1, ChREBP, and downstream lipogenic genes, thereby ameliorating hepatic steatosis in HFD-fed mice (Tang et al., 2023). Furthermore, a recent study demonstrated that, during NAFLD progression, LDHA-mediated H3K18 lactylation at the proximal promoter of METTL3 increased METTL3 expression. METTL3 subsequently stabilized SCD1 in a YTHDF1-dependent manner, thereby promoting hepatic lipid accumulation (Meng et al., 2024). Collectively, these findings indicate that specific m^6^A regulatory axes may contribute to hepatic steatosis by modulating the expression or stability of key lipogenic transcripts.

M^6^A regulation also affects lipid droplet turnover and cellular metabolic homeostasis. METTL14 expression was increased in liver tissues from patients with MASLD, a newly defined nomenclature that evolved from the former term NAFLD, and in HFD-fed mice (Zhou J. X. et al., 2024). Similarly, METTL3 was upregulated in the livers of NAFLD mice and increased m^6^A modification and YTHDF1-dependent stabilization of Rubicon mRNA. Because Rubicon inhibits autophagosome–lysosome fusion, this regulatory axis impaired lipid droplet clearance, whereas METTL3 depletion enhanced hepatic autophagic flux and lipid droplet clearance (Peng et al., 2022). Notably, m^6^A regulation may also exert protective effects under specific conditions. METTL3 increased the stability of DDIT4 mRNA through m^6^A modification, while DDIT4 reduced hepatic lipid accumulation and inflammatory activity through the mTORC1 and NF-κB signaling pathways, thereby alleviating lipid accumulation and inflammation in hepatocytes (Zhang G. et al., 2023). These findings indicate that the biological consequences of m^6^A dysregulation are not uniformly pathogenic and may vary according to the specific regulator, target transcript, and metabolic context.

Environmental exposures may further modify m^6^A-mediated regulation of hepatic lipid metabolism. NAFLD is influenced by multiple environmental and metabolic stressors, and m^6^A regulation may serve as a molecular link between these exposures and hepatic lipid metabolic dysfunction. Exposure to fine particulate matter (PM2.5) increased hepatic lipid accumulation *in vitro* and *in vivo*, accompanied by elevated global m^6^A levels and reduced YTHDC1 expression. PM2.5 exposure also altered the expression of CEPT1 and YWHAH mRNAs, potentially through YTHDC2-mediated regulation of their coding sequences (Chen et al., 2021). Arsenic exposure, another environmental risk factor for NAFLD, has been associated with disturbances in fatty acid and methionine metabolism and increased hepatic lipid accumulation. In arsenic-exposed liver tissues, increased SREBP1 and lipogenic gene expression was accompanied by reduced m^6^A and S-adenosylmethionine (SAM) levels. Mechanistically, arsenic may disrupt m^6^A-dependent maturation of miR-142-5p by depleting SAM, thereby affecting SREBP1 and promoting hepatic lipid accumulation. Supplementation with folic acid and vitamin B12 restored SAM levels and reduced hepatic lipid accumulation in arsenic-exposed mice (Yang et al., 2023a). These findings suggest that environmental factors may influence hepatic lipid metabolism through alterations in m^6^A-dependent RNA regulation, although the precise mechanisms remain to be further defined.

M^6^A dysregulation may also influence the progression of NAFLD beyond hepatic steatosis. RNA methylation regulators have been implicated in the progression of NAFLD toward NASH and hepatocellular carcinoma (HCC) (Li et al., 2024a). Notably, knockdown of METTL3 enhanced CD36-mediated free fatty acid uptake and aggravated CCL2-associated inflammatory responses, thereby promoting the progression of NAFLD to NASH (Li et al., 2021). In addition, the m6A readers IGF2BP1 and IGF2BP3 have been implicated in HCC progression, and their elevated expression has been associated with poor prognosis in patients with HCC (Gutschner et al., 2014; Zhao et al., 2025). Given the reversibility of epitranscriptomic modifications, targeting specific m^6^A regulatory axes may represent a potential therapeutic strategy for metabolic liver disease. However, current evidence indicates that the effects of m^6^A regulation in NAFLD are regulator-, target-, and context-dependent, and m^6^A should therefore not be regarded as uniformly pathogenic or protective.

### 4.3 Atherosclerosis

AS is a major pathological process underlying cardiovascular disease, and its development involves multiple pathological events, including endothelial injury, lipid accumulation, foam cell formation, inflammatory responses, and vascular smooth muscle cell (VSMC) proliferation. Sequencing analyses of mouse and human atherosclerotic plaques have revealed increased global m^6^A methylation levels, accompanied by elevated expression of METTL3 and METTL14. Bioinformatics analysis further indicated that a large number of differentially methylated genes were involved in the pathogenesis of as (Zheng et al., 2023). In contrast, Wu et al. reported significantly reduced m^6^A methylation levels in the peripheral blood and white blood cells of patients with AS and in corresponding mouse models (Wu L. et al., 2019). These findings suggest that m^6^A RNA methylation is dynamically altered in AS and may contribute to disease progression in a context-dependent manner. Table 4 summarizes the currently identified m^6^A regulatory mechanisms associated with AS.

**TABLE 4:** Effects of m^6^A regulators on AS through regulation of target transcript methylation and downstream molecular functions.

Regulator	Change	Target	Effect	Ref.
ALKBH5	↓	Bcl2	Decreased Bcl2 expression and increased TNF-α-induced vascular endothelial cell injury	Zhang et al. (2022b)
ALKBH5	↑	SPHK1	Increased SPHK1 expression maintains endothelial cell angiogenesis after acute ischemic stress	Kumari et al. (2021)
ALKBH5	↑	FOXM1	Increases FOXM1 mRNA stability and induces endothelial mesenchymal transition (FSH mechanism of action)	Li et al. (2025)
ALKBH5	↑	TXNIP	Reduced stability of TXNIP mRNA (MA improves mechanism of high glucose-induced endothelial injury)	Wang et al. (2022)
ALKBH5	↑	ACSL4	Reduced ACSL4 expression inhibits ox- LDL-induced iron apoptosis in HMEC-1 cells	Xiong et al. (2024)
FTO	↓	NR4A3	Docosahexaenoic acid (DHA) inhibits vascular endothelial cell proliferation and inflammation by inhibiting the FTO/NR4A3 axis	Huo et al. (2022)
IGF2BP1	↑	HMGB1	Promotion of HMGB1 mRNA stability, mechanism of apoptosis in vascular endothelial cells induced by hyperglycemia	Liang et al. (2023)
KIAA1429	↑	ROCK2	Upregulation of ROCK2 mRNA inhibits proliferation/migration of HUVECs	Rong et al. (2023b)
METTL3	↑	lncRNA H19	Enhancement of lncRNA H19 stability promotes endothelial cell inflammation and pyroptosis and exacerbates AS	Tang et al. (2024)
METTL3	↑	MCU	YTHDF3 recognition increases MCU translation and expression and is a major factor in human cytomegalovirus-induced apoptosis in vascular endothelial cells	Zhu et al. (2022)
METTL3	↑	MRTFA	YTHDF3 recognition promotes MRTFA mRNA translation and facilitates VSMC epistatic switching	Dong et al. (2025)
METTL3	↑	Braf	YTHDF1 binds Braf mRNA and promotes its translation, activating the ERK pathway and macrophage inflammatory response	Li et al. (2023c)
METTL3	↑	Pi3k	Increased Pi3k mRNA stability promotes phenotypic switching in vascular smooth muscle cells	Zhao et al. (2022)
METTL3	↑	JAK2	Upregulation of the JAK2/STAT3 pathway upon IGF2BP1 recognition promotes angiogenesis and AS	Dong et al. (2021)
METTL3	↑	STAT1	Activation of STAT1 signaling pathway promotes oxidized low-density lipoprotein-mediated inflammation	Li et al. (2022c)
METTL3	↑	TRAF1	Promotes TRAF1 translation and endothelial cell proliferation and adhesion	Chen et al. (2024)
METTL3	↑	NPC1L1	Promotes NPC1L1 expression and AS progression through MAPK pathway inactivation	Zhang G et al. (2023)
METTL3	↑	HDGF	Enhanced HDGF mRNA stability and protein expression promotes macrophage M1-type differentiation	Zheng L et al. (2022)
METTL3	↑	PGC-1α	YTHDF2 synergistically promotes PGC-1α mRNA degradation and increases mitochondrial dysfunction and inflammation in monocytes	Zhang et al. (2021)
METTL3	↑	hsa_circ_0029589	Inhibits circ_0029589 and promotes macrophage pyroptosis and inflammation (IRF-1 mechanism of action)	Guo et al. (2020)
METTL3	↓	AKT1S1	Promotion of AKT1S1 mRNA stability, activation of foam cell autophagy and metabolic remodeling (mechanism of action of epsilonine)	Yu et al. (2024)
METTL3	↑	UCHL1	Reduces the expression of UCHL1. Causes endothelial damage (HCMV mechanism of action)	Zhu et al. (2024)
METTL3 /METTL14	↓	NF-κB	Reduced NF-κ B mRNA stability ultimately leads to inflammatory macrophage inactivation and HTZZW alleviates AS progression mechanism	Yu et al. (2022)
METTL14	↑	miR-19a	Promoting the processing of mature miR-19a for cardiovascular endothelial cell proliferation and invasion	Zhang et al. (2020)
METTL14	↓	NEAT1	Reduced NEAT1 expression and exercise improves endothelial cell focal death mechanisms	Yang et al. (2023b)
METTL14	↑	circARHGAP12/ASPH	Upregulation of circARHGAP12 and ASPH exacerbates lipid peroxidation damage and promotes AS progression	Min et al. (2024)
METTL14	↑	CXCR4	Promotes CXCR4 mRNA stability and increases endothelial inflammation and AS	Ka et al. (2024)
METTL14	↑	p65	Increased p65 expression promotes apoptosis in HUVECs cells	Liu et al. (2022)
METTL14	↑	UCHL5	YTHDF1 recognition increases UCHL5 expression and promotes VSMC proliferation, migration, inflammation, and phenotypic switching	Yang et al. (2023c)
WTAP	↑	NLRP3	Enhanced NLRP3 mRNA stability induces macrophage apoptosis and M1 polarization to promote atherosclerosis	Luo et al. (2025)
YTHDC1	↑	circ-PIAS1-5	Promotes m^6^A-dependent nuclear export of circ-PIAS1-5 and increases lipid accumulation in foam cells	Ma et al. (2025)
YTHDF2	↑	SLC7A11	Promotion of SLC7A11 mRNA degradation and acceleration of endothelial cell iron apoptosis	Li J et al. (2024)

The arrows (↑ and ↓) indicate increases or decreases in the expression/activity of m^6^A regulators.

METTL3 has emerged as an important regulator of m^6^A modification in AS, although its effects appear to vary according to the cellular and pathological context. METTL3 and YTHDF1 cooperatively promote the translation of LRP6 and DVL1 in human umbilical vein endothelial cells (HUVECs), thereby contributing to angiogenic responses (Yao et al., 2020). Another study demonstrated that METTL3 promotes the degradation of EGFR mRNA, a molecule associated with endothelial dysfunction, thereby alleviating endothelial injury (Li B. et al., 2022). In VSMCs, however, METTL3 promotes neointimal hyperplasia by increasing global RNA m^6^A methylation and inducing phenotypic transformation. METTL3 is also involved in vascular inflammation. Zhang et al. (Zhang et al., 2021) found that METTL3 and YTHDF2 jointly regulate PGC-1α mRNA and promote its degradation, thereby contributing to ox-LDL-induced monocyte inflammation. In addition, METTL3 regulates NLRP1 and KLF4 expression in an m^6^A-dependent manner, both of which are involved in inflammatory cell adhesion (Chien et al., 2021). These findings highlight the cell type- and context-dependent functions of METTL3 in AS. Recent studies have also explored the therapeutic potential of targeting METTL3. Dong et al. (Dong et al., 2021) investigated the effects of METTL3 knockdown on AS progression using *in vivo* mouse models and chicken embryo chorioallantoic membrane (CAM) assays. They found that METTL3 knockdown attenuated AS progression by suppressing the JAK2/STAT3 signaling pathway. In another study, METTL3 silencing reduced the binding of DGCR8 to pri-miR-375, thereby limiting miR-375-3p-mediated targeting of PDK1 and ultimately contributing to plaque stabilization (Chen et al., 2023b).

METTL14 has also been reported to be upregulated in AS mice and may contribute to disease progression through effects on plaque formation, lipid deposition, iron overload, and oxidative stress. Mechanistically, METTL14-mediated m^6^A modification exacerbates lipid peroxidation in endothelial cells by increasing the expression of circARHGAP12 and ASPH, thereby promoting AS progression (Min et al., 2024). In addition, METTL14 promotes the maturation of pri-miR-19a, thereby facilitating endothelial cell proliferation and infiltration within AS lesions (Zhang et al., 2020). In the context of endothelial inflammation, METTL14 upregulates FOXO1 expression through m^6^A modification and contributes to the induction of pro-inflammatory responses (Jian et al., 2020). Tang et al. further demonstrated that METTL14 is involved in cholesterol metabolism and vascular inflammation through an m^6^A-dependent regulatory axis involving the lncRNA ZFAS1 and its downstream targets ADAM10 and RAB22A (Gong et al., 2021). Conversely, interference with METTL14 expression has been reported to exert protective effects in experimental AS. METTL14 knockdown enhanced M2 macrophage polarization through the NF-κB/IL-6 signaling pathway, reduced macrophage migration, and thereby suppressed foam cell formation (Zheng Y. et al., 2022). In addition, silencing METTL14 significantly reduced aortic plaque formation in diet-induced APOE^−/−^ mice by decreasing the m^6^A modification level of p65 transcripts (Liu et al., 2022). Collectively, these findings indicate that METTL14 may exert multifaceted effects on AS depending on the target cell, molecular target, and pathological context.

M^6^A demethylases may also contribute to the regulation of AS-related pathological processes. Deng et al. (Deng et al., 2021) demonstrated that FTO promoted neointimal formation in a rat carotid artery AS model induced by balloon injury. Huo et al. (Huo et al., 2022) established an angiotensin II (Ang II)-induced inflammatory model in VSMCs and demonstrated that the FTO/NR4A3 axis contributes to Ang II-induced VSMC proliferation and inflammation. Furthermore, ox-LDL-induced upregulation of ALKBH1 was reported to promote MIAT transcription by facilitating the binding of HIF1α to myocardial infarction-associated transcript (MIAT). Conversely, ALKBH1 downregulation inhibited ox-LDL-induced MIAT expression. These findings suggest that ALKBH1 may contribute to the regulation of atherosclerotic plaque progression through modulation of MIAT expression (Wu L. et al., 2019).

Emerging evidence suggests that ferroptosis, a form of iron-dependent lipid peroxidation-associated cell death, contributes to the pathogenesis of atherosclerosis, while m^6^A methylation may modulate this process by regulating ferroptosis-related pathways. Ferroptosis is characterized by the iron-dependent accumulation of lipid peroxides, and lipid metabolic remodeling is an important determinant of ferroptosis sensitivity (Jiang et al., 2021). Emerging studies suggest that m6A regulators may influence ferroptosis by regulating the expression, stability, or translation of genes involved in lipid metabolism and ferroptosis-related pathways (Wen et al., 2026). For example, m6A-dependent regulation of lipid metabolic regulators such as SREBF1 may alter fatty acid metabolic programs and consequently affect cellular sensitivity to ferroptosis (Dai et al., 2026). In the context of atherosclerosis, YTHDF2 has been reported to promote the degradation of SLC7A11 mRNA, thereby accelerating ferroptosis in vascular endothelial cells in cerebrovascular atherosclerosis (Li J. et al., 2024). In addition, METTL14-mediated m^6^A modification has been implicated in iron overload-induced lipid peroxidative damage in vascular endothelial cells, further supporting a potential role of m^6^A-dependent regulation in ferroptosis-associated endothelial injury during atherosclerosis (Min et al., 2024). Together, these findings suggest that m^6^A may represent an important epitranscriptomic regulatory mechanism linking lipid metabolic remodeling and ferroptosis to atherosclerotic progression. However, the available evidence remains limited, and the effects of m^6^A regulation appear to depend on the specific regulator, target transcript, and cellular context. Further studies integrating m^6^A profiling, lipidomics, iron metabolism, and functional ferroptosis assays are needed to clarify this emerging regulatory network.

## 5 Future perspectives

Despite substantial progress in elucidating the role of m^6^A modification in lipid metabolism, several important questions remain unresolved. First, the involvement of m^6^A in emerging lipid metabolic disorders beyond obesity, NAFLD/MASLD, and atherosclerosis warrants further investigation, particularly in MASH, metabolic cardiomyopathy, and metabolic kidney disease. Although these disorders share common pathological features, such as mitochondrial dysfunction, oxidative stress, and chronic inflammation, the extent to which m^6^A-mediated RNA regulation contributes to their development and progression remains poorly defined. Future studies should determine whether m^6^A represents a conserved regulatory mechanism linking lipid metabolic abnormalities across different tissues or whether its effects are predominantly tissue-, cell-, and disease-specific.

Second, the therapeutic specificity and safety of targeting m^6^A regulators remain major challenges for clinical translation. Most currently available small-molecule inhibitors and degraders targeting m^6^A regulators, including FTO and METTL3, act on RNA regulatory proteins that control multiple transcripts and cellular processes. Consequently, systemic modulation of these regulators may alter physiological RNA metabolism beyond the disease-associated pathways and potentially result in unintended biological effects. Furthermore, the consequences of targeting a particular m^6^A regulator may vary according to the target transcript, cellular context, tissue distribution, and disease stage. Therefore, future therapeutic strategies should move beyond broad modulation of m^6^A regulators toward target-, tissue-, and cell-specific interventions. Approaches such as targeted drug delivery, selective protein degradation, and modulation of disease-relevant regulator–RNA interactions may help improve therapeutic precision while minimizing off-target effects.

Third, advances in multi-omics technologies will be critical for defining the causal and context-dependent functions of m^6^A in lipid metabolic disorders. Integration of transcriptome-wide m^6^A profiling with single-cell transcriptomics, spatial transcriptomics, proteomics, and metabolomics could provide a more comprehensive view of cell- and tissue-specific m^6^A regulatory networks. In particular, longitudinal studies and functional validation in physiologically relevant models will be important for distinguishing primary m^6^A-dependent regulatory events from secondary changes resulting from metabolic stress or disease progression. Greater consideration should also be given to interindividual variation, sex, metabolic status, disease stage, and environmental exposures, all of which may influence m^6^A-mediated regulation. Such integrated approaches may facilitate the identification of disease-specific m^6^A–RNA regulatory axes and accelerate the translation of epitranscriptomic mechanisms into precision therapeutic strategies.

Overall, m^6^A represents an important regulatory layer linking RNA metabolism with lipid metabolic homeostasis. However, its biological effects are not uniform and depend on the specific regulator, target transcript, cellular environment, tissue, and disease stage. A deeper understanding of this context dependency, together with improved strategies for precise therapeutic intervention, will be essential to determine whether m^6^A can serve as a robust biomarker or therapeutic target for lipid metabolic disorders.

## References

[B1] Alarcón C. R. Goodarzi H. Lee H. Liu X. Tavazoie S. Tavazoie S. F. (2015). HNRNPA2B1 is a mediator of m^6^A-Dependent nuclear RNA processing events. Cell 162 (6), 1299–1308. 10.1016/j.cell.2015.08.011 26321680 PMC4673968

[B2] Allis C. D. Jenuwein T. (2016). The molecular hallmarks of epigenetic control. Nat. Rev. Genet. 17 (8), 487–500. 10.1038/nrg.2016.59 27346641

[B3] An Y. Duan H. (2022). The role of m6A RNA methylation in cancer metabolism. Mol. Cancer 21 (1), 14. 10.1186/s12943-022-01500-4 PMC8753874 35022030

[B4] Cai M. Liu Q. Jiang Q. Wu R. Wang X. Wang Y. (2019). Loss of m(6) A on FAM134B promotes adipogenesis in porcine adipocytes through m(6) A-YTHDF2-dependent way. IUBMB Life 71 (5), 580–586. 10.1002/iub.1974 30506811

[B5] Carletti A. Cardoso C. Lobo-Arteaga J. Sales S. Juliao D. Ferreira I. (2022). Antioxidant and anti-inflammatory extracts from sea cucumbers and tunicates induce a pro-osteogenic effect in zebrafish larvae. Front. Nutr. 9, 888360. 10.3389/fnut.2022.888360 PMC9125325 35614979

[B6] Chandel N. S. (2021). Lipid metabolism. Cold Spring Harb. Perspect. Biol. 13 (9), a040576. 10.1101/cshperspect.a040576 PMC8411952 34470787

[B7] Chang G. Shi L. Ye Y. Shi H. Zeng L. Tiwary S. (2020). YTHDF3 induces the translation of m(6)A-Enriched gene transcripts to promote breast cancer brain metastasis. Cancer Cell 38 (6), 857–871.e7. 10.1016/j.ccell.2020.10.004 33125861 PMC7738369

[B8] Chang K. Hong F. Liu H. Fang Y. Wang H. Song N. (2025). FTO aggravates podocyte injury and diabetic nephropathy progression via m6A-dependent stabilization of ACC1 mRNA and promoting fatty acid metabolism. Biochem. Pharmacol. 235, 116819. 10.1016/j.bcp.2025.116819 39988261

[B9] Chao X. Guo L. Ye C. Liu A. Wang X. Ye M. (2024). ALKBH5 regulates chicken adipogenesis by mediating LCAT mRNA stability depending on m(6)A modification. BMC Genomics 25 (1), 634. 10.1186/s12864-024-10537-2 PMC11197345 38918701

[B10] Chen J. X. Wu L. Yang G. Zhang C. Liu X. Sun X. (2021). *The influence of PM* _2.5_ *exposure on non-alcoholic fatty liver disease* . Life Sci. 270, 270. 10.1016/j.lfs.2021.119135 33513397

[B11] Chen L. Gao Y. Xu S. Yuan J. Wang M. Li T. (2023). N6-methyladenosine reader YTHDF family in biological processes: structures, roles, and mechanisms. Front. Immunol. 14, 1162607. 10.3389/fimmu.2023.1162607 PMC10043241 36999016

[B12] Chen J. Ye M. Bai J. Gong Z. Yan L. Gu D. (2023a). ALKBH5 enhances lipid metabolism reprogramming by increasing stability of FABP5 to promote pancreatic neuroendocrine neoplasms progression in an m6A-IGF2BP2-dependent manner. J. Transl. Med. 21 (1), 741. 10.1186/s12967-023-04578-6 PMC10588038 37858219

[B13] Chen J. Lai K. Yong X. Yin H. Chen Z. Wang H. (2023b). Silencing METTL3 stabilizes atherosclerotic plaques by regulating the phenotypic transformation of vascular smooth muscle cells via the miR-375-3p/PDK1 axis. Cardiovasc Drugs Ther. 37 (3), 471–486. 10.1007/s10557-022-07348-6 35704246

[B14] Chen D. Xu W. Zheng H. Zhang Y. Lin Y. Han Y. (2024). The methyltransferase METTL3 regulates endothelial cell proliferation and inflammation via m(6)A RNA methylation-mediated TRAF1 expression. Biochem. Biophys. Res. Commun. 732, 150399. 10.1016/j.bbrc.2024.150399 39033551

[B15] Chen J. Ye M. Gu D. Yu P. Xu L. Xue B. (2025). FTO-induced APOE promotes the malignant progression of pancreatic neuroendocrine neoplasms through FASN-mediated lipid metabolism. Int. J. Biol. Sci. 21 (4), 1478–1496. 10.7150/ijbs.103428 PMC11844274 39990672

[B16] Chien C. S. Li J. Y. S. Chien Y. Wang M. L. Yarmishyn A. A. Tsai P. H. (2021). METTL3-dependent N(6)-methyladenosine RNA modification mediates the atherogenic inflammatory cascades in vascular endothelium. Proc. Natl. Acad. Sci. U. S. A. 118 (7), e2025070118. 10.1073/pnas.2025070118 PMC7896299 33579825

[B17] Choe J. Lin S. Zhang W. Liu Q. Wang L. Ramirez-Moya J. (2018). mRNA circularization by METTL3-eIF3h enhances translation and promotes oncogenesis. Nature 561 (7724), 556–560. 10.1038/s41586-018-0538-8 30232453 PMC6234840

[B18] Cipriano A. Sbardella G. Ciulli A. (2020). Targeting epigenetic reader domains by chemical biology. Curr. Opin. Chem. Biol. 57, 82–94. 10.1016/j.cbpa.2020.05.006 32739717

[B19] Dai B. Li J. Xu L. Chen W. Chen J. Song M. (2026). YTHDF2-mediated stabilization of SREBF1 promotes lipid metabolic reprogramming and ferroptosis-associated radioresistance in anaplastic thyroid carcinoma. Cancer Lett. 639, 218232. 10.1016/j.canlet.2025.218232 41435981

[B20] Deng K. Ning X. Ren X. Yang B. Li J. Cao J. (2021). Transcriptome-wide N6-methyladenosine methylation landscape of coronary artery disease. Epigenomics 13 (10), 793–808. 10.2217/epi-2020-0372 33876670

[B21] Devaux Y. Nossent A. Y. (2020). A role for m6A RNA methylation in heart failure development? Eur. J. Heart Fail 22 (1), 67–69. 10.1002/ejhf.1714 31867852

[B22] Dong G. Yu J. Shan G. Su L. Yu N. Yang S. (2021). N6-Methyladenosine methyltransferase METTL3 promotes angiogenesis and atherosclerosis by upregulating the JAK2/STAT3 pathway via m6A reader IGF2BP1. Front. Cell Dev. Biol. 9, 731810. 10.3389/fcell.2021.731810 PMC8689138 34950654

[B23] Dong Z. Jin Y. Shen Y. Huang J. Tan J. Feng Q. (2025). METTL3-catalyzed m6A methylation facilitates the contribution of vascular smooth muscle cells to atherosclerosis. Cardiovasc Res. 121, 568–584. 10.1093/cvr/cvaf029 39977233

[B24] Du H. Zhao Y. He J. Zhang Y. Xi H. Liu M. (2016). YTHDF2 destabilizes m(6)A-containing RNA through direct recruitment of the CCR4-NOT deadenylase complex. Nat. Commun. 7, 12626. 10.1038/ncomms12626 27558897 PMC5007331

[B25] Gong C. Fan Y. Liu J. (2021). METTL14 mediated m6A modification to LncRNA ZFAS1/RAB22A: a novel therapeutic target for atherosclerosis. Int. J. Cardiol. 328, 177. 10.1016/j.ijcard.2020.12.002 33301833

[B26] Guo M. Yan R. Ji Q. Yao H. Sun M. Duan L. (2020). IFN regulatory Factor-1 induced macrophage pyroptosis by modulating m6A modification of circ_0029589 in patients with acute coronary syndrome. Int. Immunopharmacol. 86, 106800. 10.1016/j.intimp.2020.106800 32674051

[B27] Gutschner T. Hämmerle M. Pazaitis N. Bley N. Fiskin E. Uckelmann H. (2014). Insulin-like growth factor 2 mRNA-binding protein 1 (IGF2BP1) is an important protumorigenic factor in hepatocellular carcinoma. Hepatology 59 (5), 1900–1911. 10.1002/hep.26997 24395596

[B28] Hu Y. Feng Y. Zhang L. Jia Y. Cai D. Qian S. B. (2020). GR-mediated FTO transactivation induces lipid accumulation in hepatocytes via demethylation of m(6)A on lipogenic mRNAs. RNA Biol. 17 (7), 930–942. 10.1080/15476286.2020.1736868 32116145 PMC7549648

[B29] Huang X. T. Li J. H. Zhu X. X. Huang C. S. Gao Z. X. Xu Q. C. (2021). *HNRNPC impedes m* ^6^ *A-dependent anti-metastatic alternative splicing events in pancreatic ductal adenocarcinoma* . Cancer Lett. 518, 196–206. 10.1016/j.canlet.2021.07.016 34271104

[B30] Huang M. Guo J. Liu L. Jin H. Chen X. Zou J. (2023). m6A demethylase FTO and osteoporosis: potential therapeutic interventions. Front. Cell Dev. Biol. 11, 1275475. 10.3389/fcell.2023.1275475 PMC10667916 38020896

[B31] Huang X. He W. Fan S. Li H. Ye G. (2024). IGF2BP3-mediated enhanced stability of MYLK represses MSC adipogenesis and alleviates obesity and insulin resistance in HFD mice. Cell Mol. Life Sci. 81 (1), 17. 10.1007/s00018-023-05076-0 38196046 PMC10776757

[B32] Huo Y. B. Gao X. Peng Q. Nie Q. Bi W. (2022). Dihydroartemisinin alleviates AngII-induced vascular smooth muscle cell proliferation and inflammatory response by blocking the FTO/NR4A3 axis. Inflamm. Res. 71 (2), 243–253. 10.1007/s00011-021-01533-3 35059772

[B33] Izquierdo V. Palomera-Ávalos V. Pallàs M. Griñán-Ferré C. (2021). Resveratrol supplementation attenuates cognitive and molecular alterations under maternal high-fat diet intake: epigenetic inheritance over generations. Int. J. Mol. Sci. 22 (3), 1453. 10.3390/ijms22031453 33535619 PMC7867164

[B34] Jia G. Fu Y. Zhao X. Dai Q. Zheng G. Yang Y. (2011). N6-methyladenosine in nuclear RNA is a major substrate of the obesity-associated FTO. Nat. Chem. Biol. 7 (12), 885–887. 10.1038/nchembio.687 22002720 PMC3218240

[B35] Jian D. Wang Y. Jian L. Tang H. Rao L. Chen K. (2020). METTL14 aggravates endothelial inflammation and atherosclerosis by increasing FOXO1 N6-methyladeosine modifications. Theranostics 10 (20), 8939–8956. 10.7150/thno.45178 32802173 PMC7415798

[B36] Jiang Q. Sun B. Liu Q. Cai M. Wu R. Wang F. (2019). MTCH2 promotes adipogenesis in intramuscular preadipocytes via an m(6)A-YTHDF1-dependent mechanism. Faseb J. 33 (2), 2971–2981. 10.1096/fj.201801393RRR 30339471 PMC6516144

[B37] Jiang X. Stockwell B. R. Conrad M. (2021). Ferroptosis: mechanisms, biology and role in disease. Nat. Rev. Mol. Cell Biol. 22 (4), 266–282. 10.1038/s41580-020-00324-8 33495651 PMC8142022

[B38] Jiang A. Zhang S. Wang X. Li D. (2022). RBM15 condensates modulate m(6)A modification of STYK1 to promote tumorigenesis. Comput. Struct. Biotechnol. J. 20, 4825–4836. 10.1016/j.csbj.2022.08.068 36147665 PMC9464649

[B39] Jiang T. Xiao Y. Zhou J. Luo Z. Yu L. Liao Q. (2023). Arbutin alleviates fatty liver by inhibiting ferroptosis via FTO/SLC7A11 pathway. Redox Biol. 68, 102963. 10.1016/j.redox.2023.102963 37984229 PMC10694775

[B40] Jiao Y. Zhang J. Lu L. Xu J. Qin L. (2016). The fto gene regulates the proliferation and differentiation of pre-adipocytes *in vitro* . Nutrients 8 (2), 102. 10.3390/nu8020102 26907332 PMC4772064

[B41] Kang P. Dong P. (2024). CircMETTL14(11)S upregulated METTL14 and induced CXCR4 to aggravate endothelial inflammation and atherosclerosis. Int. Immunopharmacol. 126, 110979. 10.1016/j.intimp.2023.110979 37972448

[B42] Kang Q. Zhu X. Ren D. Ky A. MacDougald O. A. O'Rourke R. W. (2023). Adipose METTL14-Elicited N(6) -Methyladenosine promotes obesity, insulin resistance, and NAFLD through suppressing beta adrenergic signaling and lipolysis. Adv. Sci. (Weinh) 10 (28), e2301645. 10.1002/advs.202301645 PMC10558699 37526326

[B43] Kaspi A. Khurana I. Ziemann M. Connor T. Spolding B. Zimmet P. (2018). Diet during pregnancy is implicated in the regulation of hypothalamic RNA methylation and risk of obesity in offspring. Mol. Nutr. Food Res. 62 (14), e1800134. 10.1002/mnfr.201800134 29882289

[B44] Kumari R. Dutta R. Ranjan P. Suleiman Z. G. Goswami S. K. Li J. (2021). ALKBH5 regulates SPHK1-Dependent endothelial cell angiogenesis following ischemic stress. Front. Cardiovasc Med. 8, 817304. 10.3389/fcvm.2021.817304 35127873 PMC8811170

[B45] Lan J. Xu B. Shi X. Pan Q. Tao Q. (2022). WTAP-mediated N(6)-methyladenosine modification of NLRP3 mRNA in kidney injury of diabetic nephropathy. Cell Mol. Biol. Lett. 27 (1), 51. 10.1186/s11658-022-00350-8 35761192 PMC9235192

[B46] Li X. Yuan B. Lu M. Wang Y. Ding N. Liu C. (2021). The methyltransferase METTL3 negatively regulates nonalcoholic steatohepatitis (NASH) progression. Nat. Commun. 12 (1), 7213. 10.1038/s41467-021-27539-3 34893641 PMC8664922

[B47] Li K. Huang W. Wang Z. Nie Q. (2022). m(6)A demethylase FTO regulate CTNNB1 to promote adipogenesis of chicken preadipocyte. J. Anim. Sci. Biotechnol. 13 (1), 147. 10.1186/s40104-022-00795-z 36461116 PMC9716549

[B48] Li B. Zhang T. Liu M. Cui Z. Zhang Y. Liu M. (2022). RNA N(6)-methyladenosine modulates endothelial atherogenic responses to disturbed flow in mice. Elife 11, e69906. 10.7554/eLife.69906 35001873 PMC8794471

[B49] Li Z. Xu Q. Huangfu N. Chen X. Zhu J. (2022c). Mettl3 promotes oxLDL-mediated inflammation through activating STAT1 signaling. J. Clin. Lab. Anal. 36 (1), e24019. 10.1002/jcla.24019 PMC8761454 34825733

[B50] Li Y. Zhang Y. Zhang T. Ping X. Wang D. Chen Y. (2023a). Rna M(6) a methylation regulates glycolysis of beige fat and contributes to systemic metabolic homeostasis. Adv. Sci. (Weinh) 10 (25), e2300436. 10.1002/advs.202300436 PMC10477848 37407508

[B51] Li Y. Zhang D. Gao Y. Wang P. Wang Z. Zhang B. (2023b). METTL3 exacerbates insulin resistance in hepatocytes by regulating m(6)A modification of cytochrome P450 2B6. Nutr. Metab. (Lond) 20 (1), 40. 10.1186/s12986-023-00762-z PMC10502999 37710320

[B52] Li Q. Yu L. Gao A. Ren R. Zhang J. Cao L. (2023c). METTL3 (methyltransferase like 3)-Dependent N6-Methyladenosine modification on braf mRNA promotes macrophage inflammatory response and atherosclerosis in mice. Arterioscler. Thromb. Vasc. Biol. 43 (5), 755–773. 10.1161/atvbaha.122.318451 36951060

[B53] Li G. Che K. Wu J. Yang B. (2024). Construction of m6A-Related gene prediction model and subtype analysis in non-obstructive azoospermia based on bioinformatics. Am. J. Reprod. Immunol. 92 (1), e13892. 10.1111/aji.13892 38958252

[B54] Li S. Mehal W. Z. Ouyang X. (2024a). RNA modifications in the progression of liver diseases: from fatty liver to cancer. Sci. China Life Sci. 67 (10), 2105–2119. 10.1007/s11427-023-2494-x PMC11545962 38809498

[B55] Li S. Lian S. Cheng W. Zhang T. Gong X. (2024b). The role of N6-methyladenosine methyltransferase RBM15 in nonalcoholic fatty liver disease. Shock 61 (2), 311–321. 10.1097/shk.0000000000002294 38150369

[B56] Li J. Zou C. Zhang Z. Xue F. (2024). N(6)-methyladenosine (m(6)A) reader YTHDF2 accelerates endothelial cells ferroptosis in cerebrovascular atherosclerosis. Mol. Cell Biochem. 479 (7), 1853–1861. 10.1007/s11010-023-04858-1 37792239

[B57] Li P. Xiang Y. Wei J. Xu X. Wang J. Yu H. (2025). Follicle-stimulating hormone promotes EndMT in endothelial cells by upregulating ALKBH5 expression. Cell Mol. Biol. Lett. 30 (1), 41. 10.1186/s11658-025-00720-y 40186131 PMC11969750

[B58] Liang A. Liu J. Wei Y. Liao Y. Wu F. Ruan J. (2023). m(6)A reader IGF2BP1 accelerates apoptosis of high glucose-induced vascular endothelial cells in a m(6)A-HMGB1 dependent manner. PeerJ 11, e14954. 10.7717/peerj.14954 37009154 PMC10062336

[B59] Liao X. Liu J. Chen Y. Liu Y. Chen W. Zeng B. (2022). Metformin combats obesity by targeting FTO in an m(6)A-YTHDF2-dependent manner. J. Drug Target 30 (9), 983–991. 10.1080/1061186x.2022.2071906 35481401

[B60] Liao Z. Wang J. Xu M. Li X. Xu H. (2024). The role of RNA m6A demethylase ALKBH5 in the mechanisms of fibrosis. Front. Cell Dev. Biol. 12, 1447135. 10.3389/fcell.2024.1447135 PMC11362088 39220683

[B61] Liu Q. Zhao Y. Wu R. Jiang Q. Cai M. Bi Z. (2019). ZFP217 regulates adipogenesis by controlling mitotic clonal expansion in a METTL3-m(6)A dependent manner. RNA Biol. 16 (12), 1785–1793. 10.1080/15476286.2019.1658508 31434544 PMC7567449

[B62] Liu T. Wei Q. Jin J. Luo Q. Liu Y. Yang Y. (2020). The m6A reader YTHDF1 promotes ovarian cancer progression via augmenting EIF3C translation. Nucleic Acids Res. 48 (7), 3816–3831. 10.1093/nar/gkaa048 31996915 PMC7144925

[B63] Liu Y. Luo G. Tang Q. Song Y. Liu D. Wang H. (2022). Methyltransferase-like 14 silencing relieves the development of atherosclerosis via m(6)A modification of p65 mRNA. Bioengineered 13 (5), 11832–11843. 10.1080/21655979.2022.2031409 35543357 PMC9275857

[B64] Liu J. Liu Y. Chen Y. Liu Y. Huang C. Luo Y. (2024). Betaine alleviates nonalcoholic fatty liver disease (NAFLD) via a manner involving BHMT/FTO/m(6)A/PGC1α signaling. J. Nutr. Biochem. 134, 109738. 10.1016/j.jnutbio.2024.109738 39154792

[B65] Luo Y. Zhang Z. Xiang L. Zhou B. Wang X. Lin Y. (2021). Analysis of N6-Methyladenosine methylation modification in fructose-induced non-alcoholic fatty liver disease. Front. Endocrinol. (Lausanne) 12, 780617. 10.3389/fendo.2021.780617 34950107 PMC8688819

[B66] Luo G. Ai Y. Zhu T. Li J. Ren Z. (2023). FTO promoted adipocyte differentiation by regulating ADRB1 gene through m(6)A modification in hycole rabbits. Anim. Biotechnol. 34 (7), 2565–2570. 10.1080/10495398.2022.2105229 35904284

[B67] Luo X. He C. Yang B. Yin S. Li K. (2025). WTAP promotes atherosclerosis by inducing macrophage pyroptosis and M1 polarization through upregulating NLRP3. Appl. Biochem. Biotechnol. 197, 2397–2416. 10.1007/s12010-024-05106-y 39747738

[B68] Ma S. Ma F. Ding N. Xie L. Yang A. Shen J. (2025). Homocysteine promotes the pathogenesis of atherosclerosis through the Circ-PIAS1-5/miR-219a-2-3p/TEAD1 axis. Adv. Sci. (Weinh) 12 (18), e2415563. 10.1002/advs.202415563 40089857 PMC12079422

[B69] Mao Y. Dong L. Liu X. M. Guo J. Ma H. Shen B. (2019). m(6)A in mRNA coding regions promotes translation via the RNA helicase-containing YTHDC2. Nat. Commun. 10 (1), 5332. 10.1038/s41467-019-13317-9 31767846 PMC6877647

[B70] Meng J. Yan C. Liu J. (2024). LDHA- mediated histone lactylation promotes the nonalcoholic fatty liver disease progression through targeting the METTL3/YTHDF1/SCD1 m6A axis. Physiol. Res. 73 (6), 985–999. 10.33549/physiolres.935289 39903889 PMC11835221

[B71] Meyer K. D. Jaffrey S. R. (2014). The dynamic epitranscriptome: N6-methyladenosine and gene expression control. Nat. Rev. Mol. Cell Biol. 15 (5), 313–326. 10.1038/nrm3785 24713629 PMC4393108

[B72] Min X. L. Lin S. Zhao X. Zhao Q. Li Y. Li X. (2024). Mechanisms of METTL14-Mediated m6A modification in promoting iron overload-induced lipid peroxidative damage in vascular endothelial cells to aggravate atherosclerosis. J. Biochem. Mol. Toxicol. 38 (12), e70066. 10.1002/jbt.70066 39588760

[B73] Ning M. Chen L. Wang Y. Xu A. Zeng R. Zhang H. (2024). The role of FTO risk haplotype in overweight/obesity and lipid parameters-results from the central China population study. Int. J. Endocrinol. 2024, 8062791. 10.1155/2024/8062791 39483856 PMC11527535

[B74] Park S. H. Ju J. S. Woo H. Yun H. J. Lee S. B. Kim S. H. (2024). The m(6)A writer RBM15 drives the growth of triple-negative breast cancer cells through the stimulation of serine and glycine metabolism. Exp. Mol. Med. 56 (6), 1373–1387. 10.1038/s12276-024-01235-w 38825643 PMC11263342

[B75] Patil D. P. Chen C. K. Pickering B. F. Chow A. Jackson C. Guttman M. (2016). m(6)A RNA methylation promotes XIST-mediated transcriptional repression. Nature 537 (7620), 369–373. 10.1038/nature19342 27602518 PMC5509218

[B76] Peng S. Xiao W. Ju D. Sun B. Hou N. Liu Q. (2019). Identification of entacapone as a chemical inhibitor of FTO mediating metabolic regulation through FOXO1. Sci. Transl. Med. 11 (488), eaau7116. 10.1126/scitranslmed.aau7116 30996080

[B77] Peng Z. Gong Y. Wang X. He W. Wu L. Zhang L. (2022). METTL3-m(6)A-Rubicon axis inhibits autophagy in nonalcoholic fatty liver disease. Mol. Ther. 30 (2), 932–946. 10.1016/j.ymthe.2021.09.016 PMC8821937 34547464

[B78] Petrenko V. Sinturel F. Riezman H. Dibner C. (2023). Lipid metabolism around the body clocks. Prog. Lipid Res. 91, 101235. 10.1016/j.plipres.2023.101235 37187314

[B79] Pirillo A. Casula M. Olmastroni E. Norata G. D. Catapano A. L. (2021). Global epidemiology of dyslipidaemias. Nat. Rev. Cardiol. 18 (10), 689–700. 10.1038/s41569-021-00541-4 33833450

[B80] Qin Y. Li B. Arumugam S. Lu Q. Mankash S. M. Li J. (2021). m(6)A mRNA methylation-directed myeloid cell activation controls progression of NAFLD and obesity. Cell Rep. 37 (6), 109968. 10.1016/j.celrep.2021.109968 34758326 PMC8667589

[B81] Qiu T. Wu C. Yao X. Han Q. Wang N. Yuan W. (2023). AS3MT facilitates NLRP3 inflammasome activation by m(6)A modification during arsenic-induced hepatic insulin resistance. Cell Biol. Toxicol. 39 (5), 2165–2181. 10.1007/s10565-022-09703-7 35226250 PMC8882720

[B82] Quarto G. Li Greci A. Bizet M. Penning A. Primac I. Murisier F. (2025). Fine-tuning of gene expression through the Mettl3-Mettl14-Dnmt1 axis controls ESC differentiation. Cell 188 (4), 998–1018.e26. 10.1016/j.cell.2024.12.009 39826545 PMC12160003

[B83] Ramesh-Kumar D. Guil S. (2022). The IGF2BP family of RNA binding proteins links epitranscriptomics to cancer. Semin. Cancer Biol. 86 (Pt 3), 18–31. 10.1016/j.semcancer.2022.05.009 35643219

[B84] Rinella M. E. Lazarus J. V. Ratziu V. Francque S. M. Sanyal A. J. Kanwal F. (2023). A multisociety Delphi consensus statement on new fatty liver disease nomenclature. J. Hepatol. 79 (6), 1542–1556. 10.1016/j.jhep.2023.06.003 37364790

[B85] Rong H. Wang D. Wang Y. Dong C. Wang G. (2023a). YTHDF1 in tumor cell metabolism: an updated review. Molecules 29 (1), 140. 10.3390/molecules29010140 PMC10779796 38202722

[B86] Rong J. Jie Y. Zhao H. (2023b). m6A 'writer' KIAA1429 regulates the proliferation and migration of endothelial cells in atherosclerosis. Mol. Biotechnol. 65 (7), 1198–1206. 10.1007/s12033-022-00614-w PMC9734602 36463391

[B87] Ronningen T. Zeng Y. Dahl M. B. Wang J. Visnovska T. Tannæs T. M. (2025). Global mapping of RNA N(6)-methyladenosine (m(6)A) in human subcutaneous and visceral adipose tissue reveals novel targets that correlate with clinical variables of obesity. Biomark. Res. 13 (1), 146. 10.1186/s40364-025-00857-0 41225618 PMC12613671

[B88] Roth G. A. Mensah G. A. Johnson C. O. Addolorato G. Ammirati E. Baddour L. M. (2020). Global burden of cardiovascular diseases and risk factors, 1990-2019: update from the GBD 2019 study. J. Am. Coll. Cardiol. 76 (25), 2982–3021. 10.1016/j.jacc.2020.11.010 33309175 PMC7755038

[B89] Roundtree I. A. Luo G. Z. Zhang Z. Wang X. Zhou T. Cui Y. (2017). *YTHDC1 mediates nuclear export of N* ^6^ *- Methyladenosine methylated mRNAs* . Elife 6, e31311. 10.7554/eLife.31311 28984244 PMC5648532

[B90] Shen Z. Liu P. Sun Q. Li Y. Acharya R. Li X. (2021). FTO inhibits UPR(mt)-induced apoptosis by activating JAK2/STAT3 pathway and reducing m6A level in adipocytes. Apoptosis 26 (7-8), 474–487. 10.1007/s10495-021-01683-z 34212271

[B91] Sheng H. Li Z. Su S. Sun W. Zhang X. Li L. (2020). YTH domain family 2 promotes lung cancer cell growth by facilitating 6-phosphogluconate dehydrogenase mRNA translation. Carcinogenesis 41 (5), 541–550. 10.1093/carcin/bgz152 31504235

[B92] Su W. Che L. Liao W. Huang H. (2024). The RNA m(6)A writer METTL3 in tumor microenvironment: emerging roles and therapeutic implications. Front. Immunol. 15, 1335774. 10.3389/fimmu.2024.1335774 38322265 PMC10845340

[B93] Sun D. Zhao T. Zhang Q. Wu M. Zhang Z. (2021). Fat mass and obesity-associated protein regulates lipogenesis via m(6) A modification in fatty acid synthase mRNA. Cell Biol. Int. 45 (2), 334–344. 10.1002/cbin.11490 33079435

[B94] Sun L. Zhang Y. Yang B. Sun S. Zhang P. Luo Z. (2023). Lactylation of METTL16 promotes cuproptosis via m(6)A-modification on FDX1 mRNA in gastric cancer. Nat. Commun. 14 (1), 6523. 10.1038/s41467-023-42025-8 37863889 PMC10589265

[B95] Takemoto S. Nakano M. Fukami T. Nakajima M. (2021). m(6)A modification impacts hepatic drug and lipid metabolism properties by regulating carboxylesterase 2. Biochem. Pharmacol. 193, 114766. 10.1016/j.bcp.2021.114766 34536357

[B96] Tang J. Zhao X. Wei W. Liu W. Fan H. Liu X. p. (2022). METTL16-mediated translation of CIDEA promotes non-alcoholic fatty liver disease progression via m6A-dependent manner. PeerJ 10, e14379. 10.7717/peerj.14379 36518278 PMC9744165

[B97] Tang Z. Sun C. Yan Y. Niu Z. Li Y. Xu X. (2023). Aberrant elevation of FTO levels promotes liver steatosis by decreasing the m6A methylation and increasing the stability of SREBF1 and ChREBP mRNAs. J. Mol. Cell Biol. 14 (9), mjac061. 10.1093/jmcb/mjac061 36352530 PMC9951264

[B98] Tang F. Tian L. H. Zhu X. H. Yang S. Zeng H. Yang Y. Y. (2024). METTL3-mediated m6A modification enhances lncRNA H19 stability to promote endothelial cell inflammation and pyroptosis to aggravate atherosclerosis. Faseb J. 38 (20), e70090. 10.1096/fj.202401337RR 39432244 PMC11580722

[B99] Voeltz G. K. Sawyer E. M. Hajnóczky G. Prinz W. A. (2024). Making the connection: how membrane contact sites have changed our view of organelle biology. Cell 187 (2), 257–270. 10.1016/j.cell.2023.11.040 38242082 PMC11830234

[B100] Wang J. Y. Lu A. Q. (2021). The biological function of m6A reader YTHDF2 and its role in human disease. Cancer Cell Int. 21 (1), 109. 10.1186/s12935-021-01807-0 33593354 PMC7885220

[B101] Wang Y. Li Y. Toth J. I. Petroski M. D. Zhang Z. Zhao J. C. (2014). N6-methyladenosine modification destabilizes developmental regulators in embryonic stem cells. Nat. Cell Biol. 16 (2), 191–198. 10.1038/ncb2902 24394384 PMC4640932

[B102] Wang X. Sun B. Jiang Q. Wu R. Cai M. Yao Y. (2018). mRNA m(6)A plays opposite role in regulating UCP2 and PNPLA2 protein expression in adipocytes. Int. J. Obes. (Lond) 42 (11), 1912–1924. 10.1038/s41366-018-0027-z 29487348

[B103] Wang X X. Wu R. Liu Y. Zhao Y. Bi Z. Yao Y. (2020). m(6)A mRNA methylation controls autophagy and adipogenesis by targeting Atg5 and Atg7. Autophagy 16 (7), 1221–1235. 10.1080/15548627.2019.1659617 31451060 PMC7469583

[B104] Wang L. Song C. Wang N. Li S. Liu Q. Sun Z. (2020). NADP modulates RNA m(6)A methylation and adipogenesis via enhancing FTO activity. Nat. Chem. Biol. 16 (12), 1394–1402. 10.1038/s41589-020-0601-2 32719557

[B105] Wang K. Peng J. Yi C. (2021). The m(6)A consensus motif provides a paradigm of epitranscriptomic studies. Biochemistry 60 (46), 3410–3412. 10.1021/acs.biochem.1c00254 33969982

[B106] Wang J. Chen L. Qiang P. (2021). The role of IGF2BP2, an m6A reader gene, in human metabolic diseases and cancers. Cancer Cell Int. 21 (1), 99. 10.1186/s12935-021-01799-x 33568150 PMC7876817

[B107] Wang L. Fan Y. f. Li B. r. Sun W. h. Wang L. n. (2022). Maslinic acid suppresses high glucose-induced inflammation by epigenetically inhibiting TXNIP expression. Curr. Med. Sci. 42 (6), 1213–1219. 10.1007/s11596-022-2657-6 36350490

[B108] Wang M. Chao M. Han H. Zhao T. Yan W. Yang G. (2024a). Hinokiflavone resists HFD-induced obesity by promoting apoptosis in an IGF2BP2-mediated bim m(6)A modification dependent manner. J. Biol. Chem. 300 (9), 107721. 10.1016/j.jbc.2024.107721 PMC11465056 39214307

[B109] Wang Y. Tian X. Wang Z. Liu D. Zhao X. Sun X. (2024b). A novel peptide encoded by circ-SLC9A6 promotes lipid dyshomeostasis through the regulation of H4K16ac-mediated CD36 transcription in NAFLD. Clin. Transl. Med. 14 (8), e1801. 10.1002/ctm2.1801 PMC11303264 39107881

[B110] Wang W. Yan J. Han L. Zou Z. L. Xu A. L. (2024c). Silencing METTL14 alleviates liver injury in non-alcoholic fatty liver disease by regulating mitochondrial homeostasis. Biomol. Biomed. 24 (3), 505–519. 10.17305/bb.2023.9698 PMC11088893 37902450

[B111] Wei D. Sun Q. Li Y. Li C. Li X. Sun C. (2021). Leptin reduces Plin5 m(6)A methylation through FTO to regulate lipolysis in piglets. Int. J. Mol. Sci. 22 (19), 10610. 10.3390/ijms221910610 34638947 PMC8508756

[B112] WenC. JiaoY. WangZ. ShiT. WangY. XuJ. (2026). m6A methylation in ferroptosis regulation: mechanisms, targets, and therapeutic interventions. J. Adv. Res. S2090-1232(26)00046-9. 10.1016/j.jare.2026.01.021 41529766

[B113] Wu R. Liu Y. Yao Y. Zhao Y. Bi Z. Jiang Q. (2018a). FTO regulates adipogenesis by controlling cell cycle progression via m(6)A-YTHDF2 dependent mechanism. Biochim. Biophys. Acta Mol. Cell Biol. Lipids 1863 (10), 1323–1330. 10.1016/j.bbalip.2018.08.008 30305247

[B114] Wu R. Yao Y. Jiang Q. Cai M. Liu Q. Wang Y. (2018b). Epigallocatechin gallate targets FTO and inhibits adipogenesis in an mRNA m(6)A-YTHDF2-dependent manner. Int. J. Obes. (Lond) 42 (7), 1378–1388. 10.1038/s41366-018-0082-5 29795461

[B115] Wu R. Guo G. Bi Z. Liu Y. Zhao Y. Chen N. (2019). m(6)A methylation modulates adipogenesis through JAK2-STAT3-C/EBPβ signaling. Biochim. Biophys. Acta Gene Regul. Mech. 1862 (8), 796–806. 10.1016/j.bbagrm.2019.06.008 31295563

[B116] Wu L. Pei Y. Zhu Y. Jiang M. Wang C. Cui W. (2019). Association of N(6)-methyladenine DNA with plaque progression in atherosclerosis via myocardial infarction-associated transcripts. Cell Death Dis. 10 (12), 909. 10.1038/s41419-019-2152-6 31797919 PMC6892866

[B117] Wu J. Li Y. Yu J. Gan Z. Wei W. Wang C. (2020). Resveratrol attenuates high-fat diet induced hepatic lipid homeostasis disorder and decreases m(6)A RNA methylation. Front. Pharmacol. 11, 568006. 10.3389/fphar.2020.568006 33519432 PMC7845411

[B118] Wu R. Chen Y. Liu Y. Zhuang L. Chen W. Zeng B. (2021). m6A methylation promotes white-to-beige fat transition by facilitating Hif1a translation. EMBO Rep. 22 (11), e52348. 10.15252/embr.202052348 34569703 PMC8567275

[B119] Xiao W. Adhikari S. Dahal U. Chen Y. S. Hao Y. J. Sun B. F. (2016). *Nuclear m* ^6^ *A reader YTHDC1 regulates mRNA splicing* . Mol. Cell 61 (4), 507–519. 10.1016/j.molcel.2016.01.012 26876937

[B120] Xiao Y. Jiang T. Qi X. Zhou J. Pan T. Liao Q. (2025). PROTAC-mediated FTO protein degradation effectively alleviates diet-induced obesity and hepatic steatosis. Int. J. Biol. Macromol. 285, 138292. 10.1016/j.ijbiomac.2024.138292 39631579

[B121] Xiao L. De Jesus D. F. Ju C. W. Wei J. B. Hu J. DiStefano-Forti A. (2025). Divergent roles of m(6)A in orchestrating brown and white adipocyte transcriptomes and systemic metabolism. Nat. Commun. 16 (1), 533. 10.1038/s41467-024-55694-w 39788955 PMC11718074

[B122] Xie R. Yan S. Zhou X. Gao Y. Qian Y. Hou J. (2023). Activation of METTL3 promotes white adipose tissue beiging and combats obesity. Diabetes 72 (8), 1083–1094. 10.2337/db22-0775 37224383

[B123] Xiong Q. Luo Z. Xie X. Zhou W. (2024). KLF7 reverses ox-LDL-induced ferroptosis in HMEC-1 cells through transcriptionally activating ALKBH5 to inhibit the m6A modification of ACSL4. Cytotechnology 76 (6), 653–666. 10.1007/s10616-024-00641-2 39435423 PMC11490622

[B124] Yao Y. Bi Z. Wu R. Zhao Y. Liu Y. Liu Q. (2019). METTL3 inhibits BMSC adipogenic differentiation by targeting the JAK1/STAT5/C/EBPβ pathway via an m(6)A-YTHDF2-dependent manner. Faseb J. 33 (6), 7529–7544. 10.1096/fj.201802644R 30865855

[B125] Yan S. Zhou X. Wu C. Gao Y. Qian Y. Hou J. (2023). Adipocyte YTH N(6)-methyladenosine RNA-binding protein 1 protects against obesity by promoting white adipose tissue beiging in male mice. Nat. Commun. 14 (1), 1379. 10.1038/s41467-023-37100-z 36914671 PMC10011519

[B126] Yang Y. Cai J. Yang X. Wang K. Sun K. Yang Z. (2022). Dysregulated m6A modification promotes lipogenesis and development of non-alcoholic fatty liver disease and hepatocellular carcinoma. Mol. Ther. 30 (6), 2342–2353. 10.1016/j.ymthe.2022.02.021 35192934 PMC9171149

[B127] Yang Z. Yu G. l. Zhu X. Peng T. h. Lv Y. c. (2022). Critical roles of FTO-mediated mRNA m6A demethylation in regulating adipogenesis and lipid metabolism: implications in lipid metabolic disorders. Genes Dis. 9 (1), 51–61. 10.1016/j.gendis.2021.01.005 35005107 PMC8720706

[B128] Yang X. Weber A. A. Mennillo E. Secrest P. Chang M. Wong S. (2023a). Effects of early life oral arsenic exposure on intestinal tract development and lipid homeostasis in neonatal mice: implications for NAFLD development. Environ. Health Perspect. 131 (9), 97001. 10.1289/ehp12381 PMC10478510 37668303

[B129] Yang Q. Chen S. Wang X. Yang X. Chen L. Huang T. (2023b). Exercise mitigates endothelial pyroptosis and atherosclerosis by downregulating NEAT1 through N6-Methyladenosine modifications. Arterioscler. Thromb. Vasc. Biol. 43 (6), 910–926. 10.1161/atvbaha.123.319251 37078289

[B130] Yang X. Wang C. Zhu G. Guo Z. Fan L. (2023c). METTL14/YTHDF1 axis-modified UCHL5 aggravates atherosclerosis by activating the NLRP3 inflammasome. Exp. Cell Res. 427 (2), 113587. 10.1016/j.yexcr.2023.113587 37044315

[B131] Yao M. D. Jiang Q. Ma Y. Liu C. Zhu C. Y. Sun Y. N. (2020). Role of METTL3-Dependent N(6)-Methyladenosine mRNA modification in the promotion of angiogenesis. Mol. Ther. 28 (10), 2191–2202. 10.1016/j.ymthe.2020.07.022 32755566 PMC7545007

[B132] Yu Z. Zheng X. Wang C. Chen C. Ning N. Peng D. (2022). The traditional Chinese medicine hua tuo zai zao wan alleviates atherosclerosis by deactivation of inflammatory macrophages. Evid. Based Complement. Altern. Med. 2022, 2200662–2200669. 10.1155/2022/2200662 PMC8979684 35388302

[B133] Yu X. Zhang Y. Wang J. Wang X. Chen X. Yin K. (2024). Leonurine improves atherosclerosis by activating foam cell autophagy and metabolic remodeling via METTL3-mediated AKT1S1 mRNA stability modulation. Phytomedicine 134, 155939. 10.1016/j.phymed.2024.155939 39214016

[B134] Zaccara S. Ries R. J. Jaffrey S. R. (2019). Reading, writing and erasing mRNA methylation. Nat. Rev. Mol. Cell Biol. 20 (10), 608–624. 10.1038/s41580-019-0168-5 31520073

[B135] Zhang Y. Guo F. Zhao R. (2016). Hepatic expression of FTO and fatty acid metabolic genes changes in response to lipopolysaccharide with alterations in m(6)A modification of relevant mRNAs in the chicken. Br. Poult. Sci. 57 (5), 628–635. 10.1080/00071668.2016.1201199 27398647

[B136] Zhang B. Y. Han L. Tang Y. F. Zhang G. X. Fan X. L. Zhang J. J. (2020). METTL14 regulates M6A methylation-modified primary miR-19a to promote cardiovascular endothelial cell proliferation and invasion. Eur. Rev. Med. Pharmacol. Sci. 24 (12), 7015–7023. 10.26355/eurrev_202006_21694 32633395

[B137] Zhang X. Li X. Jia H. An G. Ni J. (2021). The m(6)A methyltransferase METTL3 modifies PGC-1alpha mRNA promoting mitochondrial dysfunction and oxLDL-induced inflammation in monocytes. J. Biol. Chem. 297 (3), 101058. 10.1016/j.jbc.2021.101058 34375639 PMC8406003

[B138] Zhang X. Li M. j. Xia L. Zhang H. (2022a). The biological function of m6A methyltransferase KIAA1429 and its role in human disease. PeerJ 10, e14334. 10.7717/peerj.14334 PMC9657180 36389416

[B139] Zhang C. Sun Q. Zhang X. Qin N. Pu Z. Gu Y. (2022). Gene amplification-driven RNA methyltransferase KIAA1429 promotes tumorigenesis by regulating BTG2 via m6A-YTHDF2-dependent in lung adenocarcinoma. Cancer Commun. (Lond) 42 (7), 609–626. 10.1002/cac2.12325 35730068 PMC9257983

[B140] Zhang X. Deng S. Peng Y. Wei H. Tian Z. (2022b). ALKBH5 inhibits TNF-α-induced apoptosis of HUVECs through Bcl-2 pathway. Open Med. (Wars) 17 (1), 1092–1099. 10.1515/med-2022-0484 PMC9202073 35799597

[B141] Zhang H. Yin M. Huang H. Zhao G. Lu M. (2023). METTL16 in human diseases: what should we do next? Open Med. (Wars) 18 (1), 20230856. 10.1515/med-2023-0856 38045858 PMC10693013

[B142] Zhang G. Li X. Huang X. (2023). m6A-related bioinformatics analysis and functional characterization reveals that METTL3-mediated NPC1L1 mRNA hypermethylation facilitates progression of atherosclerosis via inactivation of the MAPK pathway. Inflamm. Res. 72 (3), 429–442. 10.1007/s00011-022-01681-0 36583755

[B143] Zhao X. Yang Y. Sun B. F. Shi Y. Yang X. Xiao W. (2014). FTO-dependent demethylation of N6-methyladenosine regulates mRNA splicing and is required for adipogenesis. Cell Res. 24 (12), 1403–1419. 10.1038/cr.2014.151 25412662 PMC4260349

[B144] Zhao Y. Xia A. Li C. Long X. Bai Z. Qiu Z. (2022). Methyltransferase like 3-mediated N6-methylatidin methylation inhibits vascular smooth muscle cells phenotype switching via promoting phosphatidylinositol 3-kinase mRNA decay. Front. Cardiovasc Med. 9, 913039. 10.3389/fcvm.2022.913039 36386358 PMC9649646

[B145] Zhao L. Huang H. Luo L. Huang Z. Wu Z. Wang F. (2025). The m6A reader IGF2BP3 promotes HCC progression by enhancing MCM10 stability. Sci. Rep. 15 (1), 8204. 10.1038/s41598-025-93062-w 40065065 PMC11894129

[B146] Zheng Y. Li Y. Ran X. Wang D. Zheng X. Zhang M. (2022). Mettl14 mediates the inflammatory response of macrophages in atherosclerosis through the NF-κB/IL-6 signaling pathway. Cell Mol. Life Sci. 79 (6), 311. 10.1007/s00018-022-04331-0 35598196 PMC9124663

[B147] Zheng L. Chen X. Yin Q. Gu J. Chen J. Chen M. (2022). RNA-m6A modification of HDGF mediated by Mettl3 aggravates the progression of atherosclerosis by regulating macrophages polarization via energy metabolism reprogramming. Biochem. Biophys. Res. Commun. 635, 120–127. 10.1016/j.bbrc.2022.10.032 36265285

[B148] Zheng X. Zhou B. Li Y. Zhong H. Huang Z. Gu M. (2023). Transcriptome-wide N(6)-methyladenosine methylation profile of atherosclerosis in mice. BMC Genomics 24 (1), 774. 10.1186/s12864-023-09878-1 38097926 PMC10720251

[B149] Zhong X. Yu J. Frazier K. Weng X. Li Y. Cham C. M. (2018). Circadian clock regulation of hepatic lipid metabolism by modulation of m(6)A mRNA methylation. Cell Rep. 25 (7), 1816–1828 e4. 10.1016/j.celrep.2018.10.068 30428350 PMC6532766

[B150] Zhou Y. Hambly B. D. Simmons D. McLachlan C. S. (2021). RUNX1T1 rs34269950 is associated with obesity and metabolic syndrome. QJM 114 (8), 553–558. 10.1093/qjmed/hcaa208 32589708

[B151] Zhou J. X. Yang M. Y. Zhai D. G. Jiang Q. Zhang Q. (2024). Overexpression of METTL14 mediates steatohepatitis and insulin resistance in mice. Heliyon 10 (15), e35467. 10.1016/j.heliyon.2024.e35467 39165987 PMC11334898

[B152] Zhou B. Luo Y. Bi H. Zhang N. Ma M. Dong Z. (2024). Amelioration of nonalcoholic fatty liver disease by inhibiting the deubiquitylating enzyme RPN11. Cell Metab. 36 (10), 2228–2244.e7. 10.1016/j.cmet.2024.07.014 39146936

[B153] Zhu W. Zhang H. Wang S. (2022). Vitamin D3 suppresses human cytomegalovirus-induced vascular endothelial apoptosis via rectification of paradoxical m6A modification of mitochondrial calcium uniporter mRNA, which is regulated by METTL3 and YTHDF3. Front. Microbiol. 13, 861734. 10.3389/fmicb.2022.861734 35359726 PMC8963461

[B154] Zhu W. Zhu W. Wang S. Liu S. Zhang H. (2024). UCHL1 deficiency upon HCMV infection induces vascular endothelial inflammatory injury mediated by mitochondrial iron overload. Free Radic. Biol. Med. 211, 96–113. 10.1016/j.freeradbiomed.2023.12.002 38081437

